# Selective Cdk9 inhibition resolves neutrophilic inflammation and enhances cardiac regeneration in larval zebrafish

**DOI:** 10.1242/dev.199636

**Published:** 2021-10-26

**Authors:** Aryan Kaveh, Finnius A. Bruton, Magdalena E. M. Oremek, Carl S. Tucker, Jonathan M. Taylor, John J. Mullins, Adriano G. Rossi, Martin A. Denvir

**Affiliations:** 1 Centre for Cardiovascular Science, Queen's Medical Research Institute, University of Edinburgh, Edinburgh, EH16 4TJ, UK; 2 Centre for Inflammation Research, Queen's Medical Research Institute, University of Edinburgh, Edinburgh, EH16 4TJ, UK; 3 Department of Physics, University of Glasgow, Glasgow, G12 8QQ, UK

**Keywords:** Cardiac, Regeneration, Zebrafish, AT7519, Neutrophil, Macrophage

## Abstract

Sustained neutrophilic inflammation is detrimental for cardiac repair and associated with adverse outcomes following myocardial infarction (MI). An attractive therapeutic strategy to treat MI is to reduce or remove infiltrating neutrophils to promote downstream reparative mechanisms. CDK9 inhibitor compounds enhance the resolution of neutrophilic inflammation; however, their effects on cardiac repair/regeneration are unknown. We have devised a cardiac injury model to investigate inflammatory and regenerative responses in larval zebrafish using heartbeat-synchronised light-sheet fluorescence microscopy. We used this model to test two clinically approved CDK9 inhibitors, AT7519 and flavopiridol, examining their effects on neutrophils, macrophages and cardiomyocyte regeneration. We found that AT7519 and flavopiridol resolve neutrophil infiltration by inducing reverse migration from the cardiac lesion. Although continuous exposure to AT7519 or flavopiridol caused adverse phenotypes, transient treatment accelerated neutrophil resolution while avoiding these effects. Transient treatment with AT7519, but not flavopiridol, augmented wound-associated macrophage polarisation, which enhanced macrophage-dependent cardiomyocyte number expansion and the rate of myocardial wound closure. Using *cdk9^−/−^* knockout mutants, we showed that AT7519 is a selective CDK9 inhibitor, revealing the potential of such treatments to promote cardiac repair/regeneration.

## Abstract (precis)

**Summary:** This study is the first to show that resolving neutrophilic inflammation using a clinically approved immunomodulatory drug (AT7519) improves heart regeneration in zebrafish.

## INTRODUCTION

Myocardial infarction (MI) is a leading cause of morbidity and mortality worldwide. MI occurs when a coronary artery becomes occluded, leading to myocardial ischaemia and extensive cardiomyocyte death. The surviving myocardium subsequently undergoes compensatory remodelling and scarring, which often results in secondary complications, such as heart failure. Although MI can be successfully treated and managed (Anderson and Morrow, 2017), there are no approved therapies that promote repair of the damaged myocardium. Recent clinical trials have investigated immunomodulatory therapies that inhibit pleiotropic inflammatory pathways (Ridker et al., 2017; Tardif et al., 2019). These treatments lower the incidence of cardiovascular events post-MI but increase the risk of infections. Therefore, there is a need to explore treatments that specifically target myocardial inflammation and promote downstream cardiac repair mechanisms following MI.

Neutrophils are the first immune cell recruited to the myocardial infarct, where they phagocytose dead and dying cells (Dewald et al., 2004; Swirski and Nahrendorf, 2013). Neutrophils subsequently secrete inflammatory mediators to recruit monocytes, which later differentiate into macrophages (Dewald et al., 2005; Nahrendorf et al., 2007). Once the acute inflammatory response starts to resolve, most infiltrating neutrophils undergo apoptosis (Daseke et al., 2019). Apoptotic neutrophils are efferocytosed by inflammatory macrophages, triggering a series of anti-inflammatory pathways that promote cardiac repair (Savill et al., 2002; Schwab et al., 2007; Frangogiannis and Rosenzweig, 2012; Ma et al., 2013). Conversely, defective clearance of neutrophils augments inflammation, promoting cardiomyocyte apoptosis, infarct expansion and adverse structural remodelling (Frangogiannis et al., 2002; Vinten-Johansen, 2004; Garlichs et al., 2004; van Hout et al., 2015; Schloss et al., 2016). Indeed, blood neutrophilia is recognised as an indicator of adverse clinical outcomes following MI (Arruda-Olson et al., 2009; Chia et al., 2009). Removing cardiac-recruited neutrophils therefore has potential as a viable therapeutic strategy to improve myocardial repair post-MI.

Extensive work from our group and others has shown that cyclin-dependent kinase 9 (CDK9) inhibitor compounds selectively induce neutrophil apoptosis, reduce neutrophil infiltration, and promote the resolution of inflammation *in vitro* and *in vivo* (Rossi et al., 2006; Loynes et al., 2010; Leitch et al., 2012; Wang et al., 2012; Lucas et al., 2014; Hoodless et al., 2016). Unlike most other CDKs, CDK9 specifically regulates the transcription of primary inflammatory response genes via RNA polymerase II. These include genes encoding inflammatory cytokines and the neutrophil pro-survival protein Mcl1 (Sundar et al., 2021; Eyvazi et al., 2019; Lucas et al., 2014). Acute inhibition of CDK9 therefore provides a therapeutic opportunity to suppress the transcription of short-lived inflammatory disease drivers preferentially. However, owing to the conserved structure of CDKs, CDK9 inhibitor compounds may also target other kinases (Kryštof et al., 2012). Two potent CDK9 inhibitors, AT7519 and flavopiridol (FVP), have been widely used in clinical trials as anti-cancer therapies (Mahadevan et al., 2011; Chen et al., 2014; Luke et al., 2012; Awan et al., 2016). Our group has shown that AT7519 and FVP drive neutrophil apoptosis in a CDK9-dependent manner to resolve inflammation following tail fin transection in larval zebrafish (Hoodless et al., 2016). It is not yet understood how CDK9 inhibitors influence inflammatory and repair/regeneration responses following tissue wounding.

The zebrafish has proven to be an essential model for studying cardiac injury, repair and regeneration. Unlike adult mammalian hearts, zebrafish hearts regenerate rapidly following injury via cardiomyocyte proliferation (Poss et al., 2002; Jopling et al., 2010; Kikuchi et al., 2010). Adult zebrafish cardiac injury and regeneration studies have found that sustained neutrophil retention inhibits cardiomyocyte proliferation, promotes cardiomyocyte apoptosis and delays scar regression (Lai et al., 2017; Xu et al., 2019). The resolution of neutrophilic inflammation is therefore considered a prerequisite for timely and complete heart regeneration. We recently characterised neutrophil and macrophage migratory responses in larval zebrafish cardiac injury using bespoke live imaging (Taylor et al., 2019; Kaveh et al., 2020). We identified a conserved sequence of events marked by an early and acute phase of neutrophil recruitment followed by sustained macrophage recruitment (Kaveh et al., 2020). Importantly, the dynamics of the immune cell response in larval zebrafish closely recapitulates that of adult zebrafish and murine models of cardiac injury (Bevan et al., 2020; Epelman et al., 2015).

In this study, we use our established larval zebrafish cardiac injury model to investigate whether CDK9 inhibitor (CDK9i) treatment with AT7519 or FVP resolves neutrophil infiltration and examine whether this regulates downstream macrophage involvement and cardiac regeneration. We found that both AT7519 and FVP resolved neutrophilic inflammation via reverse migration. However, subsequent drug exposure caused adverse effects, which were avoided by shortening CDK9i treatment duration. Interestingly, transient (pulsed) treatment with AT7519, but not FVP, enhanced *tnf* expression in wound-associated macrophages, in turn promoting macrophage-dependent cardiomyocyte number expansion and the rate of myocardial wound closure. We show that, unlike FVP, AT7519 is a selective CDK9 inhibitor and thus a promising immunomodulatory treatment that could promote cardiomyocyte regeneration.

## RESULTS

### CDK9i treatment resolves neutrophil infiltration by promoting reverse migration from the cardiac injury site

**Fig. 1.**
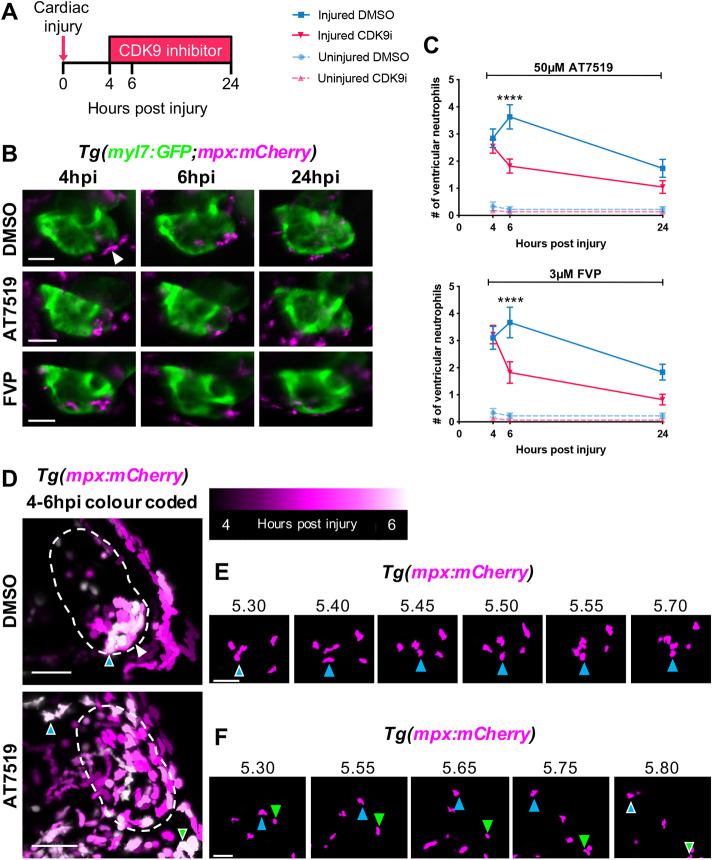
**CDK9i treatment resolves neutrophil infiltration following cardiac injury by promoting reverse migration.** (A) Experimental timeline indicating cardiac injury, CDK9i treatment and imaging time points. (B) Epifluorescence images of *Tg(myl7:GFP;mpx:mCherry)* larvae displaying neutrophil presence on the injured ventricle at 4 hpi (prior to treatment), and at 6 hpi and 24 hpi with 0.3% DMSO vehicle (top), 50 μM AT7519 (middle) or 3 μM FVP (bottom). Arrowhead indicates ventricular apex injury site marked by a loss of myocardial GFP and neutrophil accumulation. (C) Number of ventricular neutrophils at 4 hpi, 6 hpi and 24 hpi with 50 μM AT7519 (top) or 3 μM FVP (bottom) treatment. Error bars represent s.e.m., *n*=19 larvae, experimental *n*=3. ****P<0.0001 (two-way ANOVA and Bonferroni post-hoc test for comparisons between cardiac-injured DMSO vehicle or CDK9i treatment groups). (D) LSFM images of neutrophil (*mpx:mCherry*) migration temporally colour coded between 4 hpi and 6 hpi with 0.1% DMSO vehicle (top) or 50 μM AT7519 (bottom). Neutrophil positions appear as a different colour depending on the point in time (as indicated in the key). Dashed line indicates outline of ventricle. Coloured arrowheads indicate starting position of neutrophil (DMSO vehicle, blue arrowhead with white outline) or ending position of neutrophils (AT7519, blue and green arrowheads with white outline) relative to image panels in E and F, respectively. White arrowhead indicates ventricular apex injury site. (E) LSFM time-lapse-derived images of ventricular neutrophil migration with DMSO vehicle (0.1%); hpi indicated above each image. Blue arrowhead tracks an individual neutrophil migrating across the ventricular apex. (F) LSFM time-lapse-derived images of neutrophil migration from ventricle to pericardium with AT7519 (50 μM) treatment; hpi indicated above each image. Blue and green arrowheads track individual neutrophils reverse migrating anteriorly and posteriorly to the pericardium, respectively. LSFM fluorescence images were acquired in 3D and maximum intensity projections were used for temporal colour code analysis (D) or are individually displayed (E,F). Scale bars: 50 μm.

We have previously characterised cardiac injury, neutrophil recruitment and resolution dynamics following ventricular laser injury in larval zebrafish. We found that peak neutrophil infiltration occurs at 6 h post-injury (hpi) and neutrophil numbers entirely resolve by 48 hpi (Kaveh et al., 2020). Two CDK9 inhibitors, AT7519 and flavopiridol (FVP), have been shown to resolve wound-recruited neutrophil numbers by inducing apoptosis following larval zebrafish tail fin transection (Hoodless et al., 2016). To avoid disrupting the onset of inflammation in our cardiac injury model, and encourage the resolution of peak neutrophilic inflammation, *Tg(myl7:GFP;mpx:mCherry)* larvae were treated continuously with AT7519 or FVP from 4 hpi. Larvae were subsequently imaged at 6 hpi and 24 hpi using epifluorescence microscopy to quantify ventricular neutrophil numbers (Fig. 1A). Following recruitment to the injured ventricular apex at 4 hpi, neutrophil numbers increased in DMSO vehicle-treated larvae at 6 hpi (Fig. 1B,C). In contrast, fewer ventricular neutrophils were present in larvae treated with 50 μM AT7519 or 3 μM FVP at 6 hpi (1.8±0.3 versus 3.6±0.5 and 1.8±0.4 versus 3.7±0.6) (Fig. 1B,C). Neutrophil presence decreased in all groups by 24 hpi, indicating that neutrophil numbers had mostly resolved (Fig. 1B,C). To determine whether this drug-induced reduction in cardiac-neutrophil numbers was due to cell death or reverse migration, time-lapse images were acquired using heartbeat-synchronised light-sheet fluorescence microscopy (LSFM) (Taylor et al., 2019). Live imaging demonstrated that recruited neutrophils cluster specifically at the cardiac injury site in DMSO vehicle-treated larvae (Movie 1), as previously shown (Kaveh et al., 2020). This is displayed in Fig. 1D where neutrophil positions are temporally colour-coded between 4 hpi and 6 hpi, allowing neutrophil migration to be schematically summarised. Following treatment with AT7519 (Movie 2) or FVP (Movie 3), recruited neutrophils appeared to migrate more erratically, and subsequently reverse migrated to the pericardium anteriorly or posteriorly from the ventricle by 6 hpi (Fig. 1D,F). These data demonstrate that CDK9i drug treatment accelerates the resolution of peak neutrophilic inflammation at the cardiac injury site dynamically by reverse migration. As our time-lapse imaging can account for every wound-recruited immune cell (Kaveh et al., 2020), these findings exclude neutrophil apoptosis or the efferocytosis of apoptotic neutrophils as a resolution mechanism with CDK9i treatment in this model.

### Continuous CDK9i treatment reduces macrophage retention by promoting reverse migration from the injured heart

**Fig. 2.**
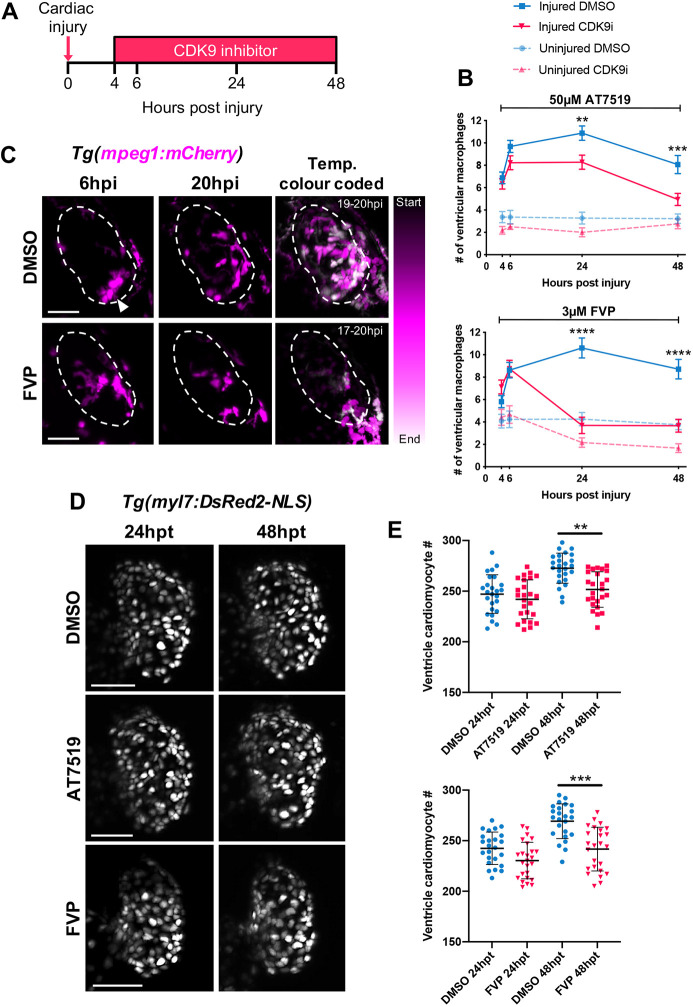
**Continuous CDK9i treatment reduces macrophage retention on the injured ventricle and impairs cardiomyocyte number expansion.** (A) Experimental timeline indicating cardiac injury, continuous CDK9i treatment and imaging time points. (B) Number of ventricular macrophages at 4 hpi, 6 hpi, 24 hpi and 48 hpi with ≤0.3% DMSO vehicle, 50 μM AT7519 (top) or 3 μM FVP (bottom) treatment. Error bars represent s.e.m., *n*=16 larvae, experimental *n*=3. **P<0.01, ***P<0.001, ****P<0.0001 (two-way ANOVA and Bonferroni post-hoc test for comparisons between cardiac-injured DMSO vehicle or CDK9i treatment groups). (C) LSFM time-lapse-derived images of cardiac-injured *Tg(mpeg1:mCherry)* larvae displaying ventricular macrophage presence at 6 hpi (left panel) and 20 hpi (middle panel) with 0.3% DMSO vehicle or 3 μM FVP. LSFM time-lapse images of macrophage (*mpeg1:mCherry*) migration temporally colour-coded with DMSO vehicle (0.3%) or FVP (3 μM) treatment (right). Start and end timepoint (hpi) for colour code is indicated. Dashed line indicates outline of ventricle. Arrowhead indicates ventricular apex injury site. (D) LSFM images of *Tg(myl7:DsRed2-NLS)* larvae displaying ventricular cardiomyocytes at 24 h hpt and 48 hpt with 0.3% DMSO vehicle (top), 50 μM AT7519 (middle) or 3 μM FVP (bottom). (E) Number of ventricular cardiomyocytes at 24 hpt and 48 hpt with ≤0.3% DMSO vehicle, 50 μM AT7519 (top) or 3 μM FVP (bottom). Error bars represent s.d., *n*=25 larvae, experimental *n*=3. **P<0.01, ***P<0.001 (one-way ANOVA and Tukey post-hoc test performed for comparisons between DMSO vehicle or CDK9i treatment groups). LSFM fluorescence images were acquired in 3D and maximum intensity projections are used for time point display (C,D) or temporal colour code analysis (C). Scale bars: 50 μm.

Having established that CDK9i treatment induces neutrophil reverse migration following cardiac injury, macrophage involvement was next examined. We have previously described macrophage recruitment dynamics in this model. Unlike the neutrophil response, macrophage recruitment occurs up to 24 hpi, with their numbers decreasing but not returning to baseline by 48 hpi (Kaveh et al., 2020). To test the effect of CDK9i treatment during the macrophage response to cardiac injury, *Tg(myl7:GFP;mpeg1:mCherry)* larvae were treated continuously with AT7519 or FVP from 4 hpi and subsequently imaged at 6 hpi, 24 hpi and 48 hpi (Fig. 2A). In the presence of AT7519 or FVP, ventricular macrophage numbers were unaffected until 24 hpi, at which point significantly fewer macrophages were present with AT7519 (8.3±0.6 versus 10.9±0.7) or FVP (3.7±0.7 versus 10.6±0.9) (Fig. 2B). This attenuated macrophage presence was more pronounced with FVP, with macrophage numbers as low as uninjured larvae (Fig. 2B). At 48 hpi, ventricular macrophage numbers remained diminished with FVP treatment (3.7±0.6 versus 8.7±0.9); similarly, AT7519-treated larvae displayed a further decrease in recruited macrophage numbers (4.9±0.6 versus 8.0±0.8) (Fig. 2B). LSFM time-lapse imaging indicated that cardiac-recruited macrophages gradually undergo reverse migration in the presence of FVP, as opposed to being retained on the injured ventricle in control conditions (Movie 4, Movie 5, Fig. 2C).

### Continuous CDK9i treatment disrupts cardiac function and cardiomyocyte number expansion

While examining the macrophage response with CDK9i treatment, it became apparent that ventricular contractility was being compromised at the later time points. Ventricular ejection fraction was diminished with FVP at 24 hpi and 48 hpi, and with AT7519 at 48 hpi, in both uninjured and cardiac-injured larvae (Fig. S1). By contrast, DMSO vehicle-treated cardiac-injured larvae displayed a complete functional recovery of ejection fraction by 48 hpi (Fig. S1). Similar to ejection fraction, heart rate was also significantly reduced with FVP at 48 hpi in uninjured and injured larvae (Fig. S1). To identify whether this loss in cardiac function is associated with a change in cardiomyocyte numbers, ventricular cardiomyocyte nuclei were quantified using *Tg(myl7:DsRed2-NLS)* larvae and LSFM. No differences were observed between groups at 24 h post-treatment (hpt). At 48 hpt, however, fewer ventricular cardiomyocytes were observed in the presence of AT7519 (251.6±17.5 versus 272.7±14.9) or FVP (241.7±21.6 versus 269.3±17.3) compared with DMSO vehicle (Fig. 2D,E). These data indicate that continuous CDK9i treatment suppresses cardiac function and the expansion of cardiomyocyte numbers.

### Continuous CDK9i treatment does not affect global macrophage numbers but causes neutropenia

Having identified undesirable cardiac-specific effects with continuous CDK9i treatment, we tested whether these compounds altered whole-body macrophage or neutrophil numbers by serially imaging *Tg(mpeg1:mCherry)* or *Tg(mpx:mCherry)* larvae. Across all experimental time points and treatment groups, whole-body macrophage numbers were unaffected and increased steadily, as expected with normal development (Fig. S2). However, at 48 hpi, significantly fewer neutrophils were present globally in the presence of AT7519 (253.7±17.7 versus 318.1±18.8) or FVP (218.8±15.9 versus 280.9±8.7) compared with their DMSO vehicle-treated counterparts (Fig. S2). Closer examination of CDK9i-treated neutropenic larvae showed that some neutrophils appear condensed and rounded – two properties of an apoptotic cell (Fig. S1). Thus, these data suggest that continuous exposure to CDK9 inhibitors promotes neutrophil but not macrophage cell death, corroborating previous studies (Lucas et al., 2014; Hoodless et al., 2016).

### Transient CDK9i treatment resolves neutrophilic inflammation without causing neutropenia or impairing cardiac contractility

To avoid the adverse cardiac effects and neutropenia apparent at the later time points with CDK9i treatment, the duration of treatment was modified. We previously showed that peak neutrophilic inflammation at 6 hpi resolves by treating larvae with AT7519 or FVP from 4 hpi (Fig. 1). Therefore, a shorter treatment was adopted whereby larvae were specifically treated with AT7519 or FVP for 2 h from 4 hpi (Fig. S3). We first confirmed that cardiac-recruited neutrophil numbers were reduced following transient (pulsed) CDK9i treatment (Fig. S3). Unlike continuous CDK9i treatment, the transient treatment was not associated with neutropenia (Fig. S3). We next assessed ventricular ejection fraction as this was noticeably diminished during continuous CDK9i treatments (Fig. S1). Following transient CDK9i treatment with AT7519 or FVP, ejection fraction recovered promptly, with injured treatment groups displaying no differences across all time points (Fig. S3).

### Transient CDK9i treatment retains cardiac macrophage numbers following injury and AT7519 enhances wound macrophage *tnf* expression

**Fig. 3.**
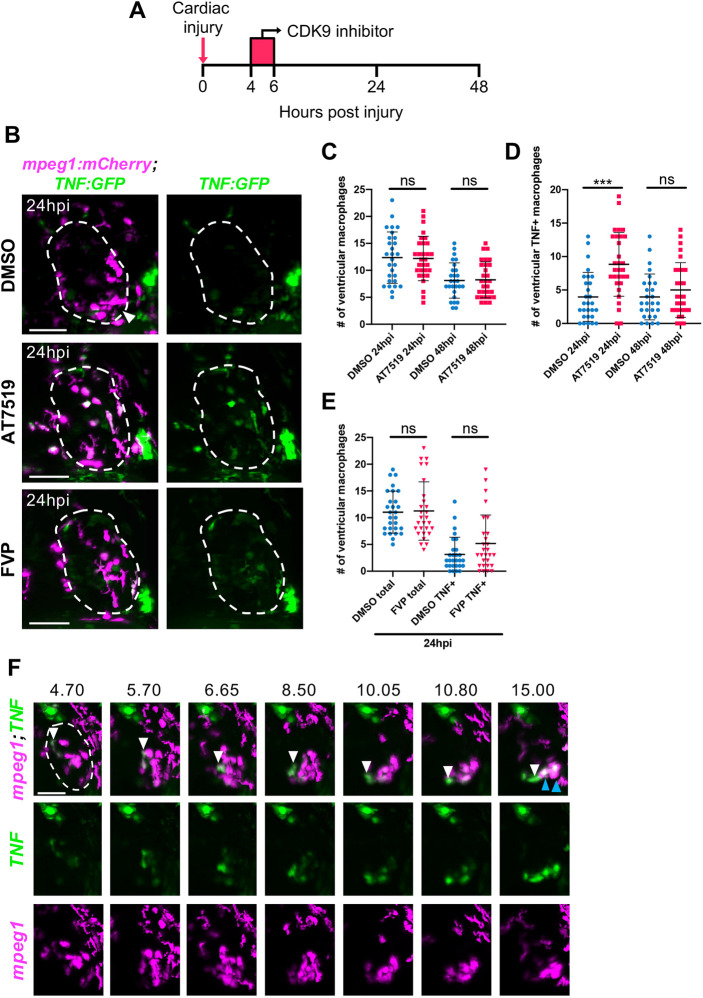
**Transient CDK9i treatment does not affect cardiac macrophage numbers and AT7519 enhances wound macrophage *tnf* polarisation following injury.** (A) Experimental timeline indicating cardiac injury, transient CDK9i treatment and imaging time points. (B) LSFM images of *Tg(mpeg1:mCherry;TNFa:GFP)* larvae displaying macrophage accumulation and *tnf* expression on the injured ventricle at 24 hpi following transient treatment with 0.3% DMSO vehicle (top), 50 μM AT7519 (middle) or 3 μM FVP (bottom). Arrowhead indicates ventricular apex injury site. (C,D) Number of ventricular macrophages (C) and ventricular *tnf*^+^ macrophages (D) at 24 hpi and 48 hpi following transient AT7519 (50 μM) treatment. (E) Number of ventricular macrophages (total and *tnf*^+^) at 24 hpi following transient FVP (3 μM) treatment. (C-E) Error bars represent s.d., *n*=28 larvae, experimental *n*=3. ***P<0.001 (one-way ANOVA and Tukey post-hoc test for comparisons between treatment groups). ns, non-significant. (F) LSFM time-lapse-derived images displaying macrophage migration and *tnf* expression on the injured ventricle, hpi indicated above each image. White arrowhead (top, all time points) tracks an individual *tnf*^+^ macrophage (*mpeg1^low^*) migrating to the ventricular apex. Blue arrowheads (top, 15.00 hpi) indicate two macrophages that have upregulated their *tnf* expression at the injured ventricular apex. LSFM fluorescence images were acquired in 3D and maximum intensity projections are displayed. Dashed line indicates outline of ventricle. Scale bars: 50 μm.

As macrophages are essential for complete myocardial repair (Ma et al., 2018), we next tested whether the revised transient CDK9i treatment (Fig. 3A) alters ventricular macrophage wound accumulation and/or polarisation. In contrast to mammalian models, only one macrophage polarisation marker has been reliably reported in larval zebrafish and this is TNF. These studies revealed *tnf*^+^ macrophages to have pro-regenerative properties following spinal cord, somitic muscle and tail fin injury (Cavone et al., 2021; Tsarouchas et al., 2018; Gurevich et al., 2018; Nguyen-Chi et al., 2017). As such, the hearts of *Tg(mpeg1:mCherry;TNFa:GFP)* larvae were analysed using LSFM following cardiac injury. Unlike continuous CDK9i treatment, ventricular macrophage retention was unaffected following transient treatment with AT7519 or FVP (Fig. 3B,C,E). Furthermore, following transient AT7519 treatment, a significant increase in ventricular *tnf*^+^ macrophages was observed at 24 hpi compared with their DMSO vehicle-treated counterparts (8.8±4.8 versus 4.0±3.7), which returned to baseline at 48 hpi (5.0±4.1 versus 4.0±3.4) (Fig. 3B,D). Interestingly, this effect was not detected following transient FVP treatment, as no statistical difference was found in ventricular *tnf*^+^ macrophage numbers at 24 hpi (5.2±5.3 versus 3.2±3.2) (Fig. 3B,E). The increase in *tnf*^+^ cardiac macrophages following transient AT7519 treatment was not observed when applied to uninjured larvae (Fig. S4), suggesting this effect to be injury specific. LSFM time-lapse imaging demonstrated that *tnf*^+^ macrophages can migrate from the pericardium onto the injured ventricle, or, more commonly, that wound-proximal macrophages upregulate *tnf* (Movie 6, Fig. 3F). Together, these data indicate that transient CDK9i treatment does not affect cardiac macrophage wound accumulation, and AT7519 enhances *tnf* expression in wound-associated macrophages.

### AT7519 is a more selective CDK9 inhibitor than FVP in zebrafish

**Fig. 4.**
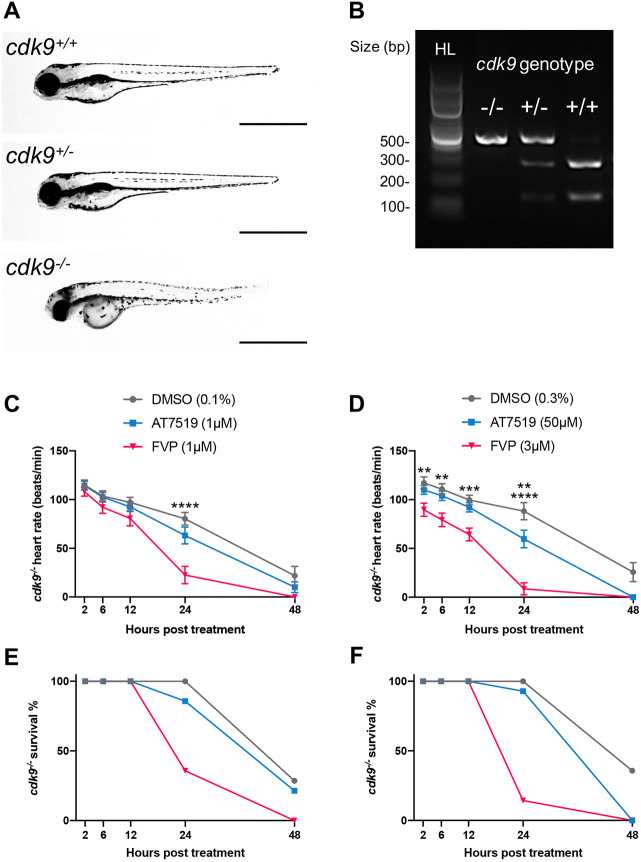
**AT7519 is a more selective CDK9 inhibitor than FVP in zebrafish.** (A) Brightfield images of a *cdk9^+/+^* (top), *cdk9^+/−^* (middle) and *cdk9^−/−^* (bottom) whole zebrafish at 4 dpf. Scale bars: 1 mm. (B) Restriction enzyme digest gel displaying *cdk9* genotypes of zebrafish larvae. Hyperladder (HL) band and individual genotype bands (in order: *cdk9^−/−^*, *cdk9^+/−^* and *cdk9^+/+^*) are indicated. (C,E) Heart rate (beats/min) (C) and percentage survival (E) of *cdk9^−/−^* larvae at 2 hpt, 6 hpt, 12 hpt, 24 hpt and 48 hpt with 0.1%, DMSO vehicle, 1 μM AT7519 or 1 μM FVP treatment. (D,F) Heart rate (beats/min) (D) and percentage survival (F) of *cdk9^−/−^* larvae at 2 hpt, 6 hpt, 12 hpt, 24 hpt and 48 hpt with 0.3% DMSO vehicle, 50 μM AT7519 or 3 μM FVP treatment. (C,D) Error bars represent s.e.m., *n*=15 larvae, experimental *n*=3. **P<0.01, ***P<0.001, ****P<0.0001 (two-way ANOVA and Bonferroni post-hoc test for comparisons between DMSO vehicle or CDK9i treatment groups).

To understand better the differential phenotypes observed with AT7519 and FVP treatment, we next explored the selectivity of these two CDK9 inhibitor compounds in larval zebrafish. Drug screening studies *in vitro* have suggested that AT7519 is a more selective CDK9 inhibitor compared with first-generation CDK9 inhibitors such as FVP (Santo et al., 2010; Liu et al., 2012). We formally tested the Cdk9 selectivity of these inhibitors *in vivo* using stable *cdk9* knockout zebrafish generated using CRISPR/Cas9 (Hoodless et al., 2016). Homozygous *cdk9* mutant zebrafish larvae are phenotypically distinguishable at 3 days post-fertilisation (dpf) (Hoodless et al., 2016). Compared with their heterozygous and wild-type siblings, homozygous *cdk9* mutants display a curved body axis, shorter body length and smaller eye diameter (Fig. 4A). No phenotypic differences were identified between heterozygous mutants and wild-type siblings up to 5 dpf (Fig. 4A); the genetic identity of these larvae was confirmed by genotyping (Fig. 4B). We reasoned that a truly selective CDK9 inhibitor would not have any effect on *cdk9^−/−^* knockout zebrafish larvae. To test this, we treated 3 dpf homozygous *cdk9* mutants continuously with DMSO vehicle, AT7519 or FVP and quantified heart rate between treatments as a readout for overall health over a 48 h time period. We have shown that continuous CDK9i treatment causes wild-type larvae to develop bradycardia (Fig. S1) and heart rate is a recognised readout of drug-induced toxicity in larval zebrafish (Rubinstein, 2006; Kithcart and MacRae, 2017). Thus, a decline in heart rate with AT7519 or FVP compared with vehicle would suggest that the compounds are acting in a Cdk9-independent manner. First, larvae were treated with 1 μM concentrations of AT7519 or FVP (or DMSO vehicle) so that CDK9i treatments were fair and comparable. Between 2 hpt and 48 hpt, all treatment groups displayed a gradual reduction in heart rate, which was associated with decreased survival from 24 hpt (Fig. 4C,E). At 24 hpt, compared with the DMSO vehicle group, FVP-treated, but not AT7519-treated, mutant larvae displayed a significant reduction in heart rate (22.5±8.9 versus 80.4±6.6 and 63.2±8.6 versus 80.4±6.6, respectively), which was associated with increased mortality (64.3% versus 14.3%) (Fig. 4C,E). The heart rates of larvae treated with AT7519 displayed a more gradual reduction, similar to their DMSO vehicle-treated counterparts (Fig. 4C,E). To draw direct comparisons between drug selectivity and the differential phenotypes observed, we applied the drug concentrations established originally for resolving neutrophilic inflammation (50 μM for AT7519 and 3 μM for FVP). Using these concentrations, FVP-treated mutant larvae displayed significantly lowered heart rates from 2 hpt (90.0±6.7 versus 117.1±6.2) until 12 hpt (64.3±6.5 versus 99.6±4.9) compared with their DMSO vehicle-treated and AT7519-treated counterparts, which showed no differences across these time points (Fig. 4D). Until 12 hpt, this FVP-induced reduction in heart rate was not associated with a change in survival (Fig. 4F). At 24 h after FVP treatment, 14% of larvae survived, all of which displayed diminished heart rates (Fig. 4D,F). In contrast, 24 h after AT7519 treatment survival only decreased to 93%, but the heart rates of these larvae were lower compared with their DMSO vehicle-treated counterparts (60.0±9.0 versus 88.2±8.7) (Fig. 4D,F). At 48 hpt, no larvae that were treated with FVP or AT7519 survived, whereas 36% of DMSO vehicle-treated survived (Fig. 4D,F). In summary, we have developed a proof-of-concept assay using knockout larval zebrafish mutants to examine drug selectivity *in vivo*. The assay indicated whether AT7519 and FVP exhibit Cdk9-independent effects up to 48 hpt, with FVP displaying significant off-target effects from 2 hpt. These comparative zebrafish data suggest that AT7519 is a particularly selective CDK9 inhibitor *in vivo*.

### Transient AT7519 but not FVP treatment enhances cardiomyocyte number expansion following injury

**Fig. 5.**
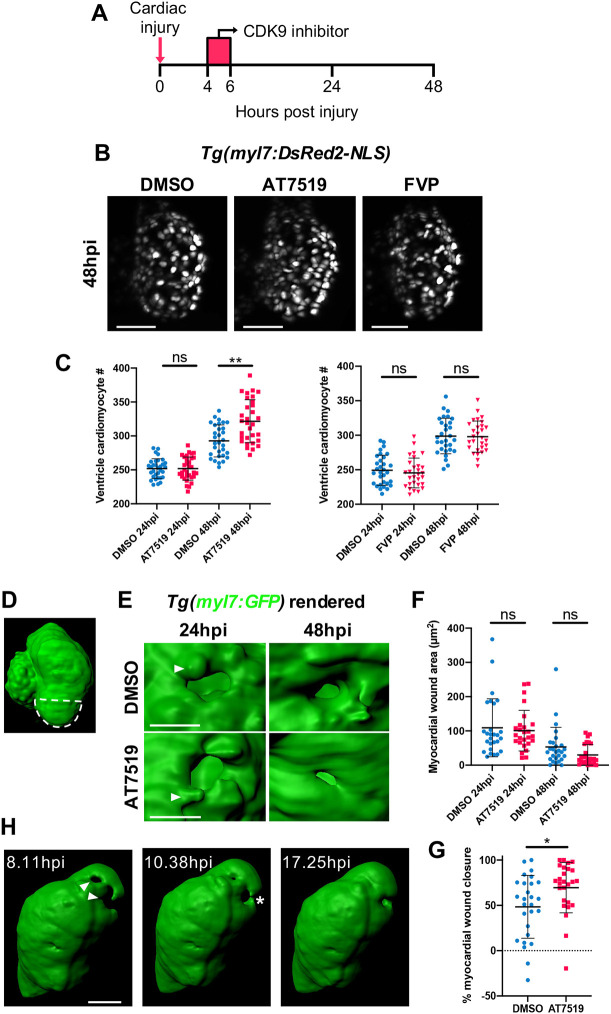
**Transient AT7519 treatment enhances cardiomyocyte number expansion and accelerates the rate of myocardial regeneration following injury.** (A) Experimental timeline indicating cardiac injury, transient CDK9i treatment and imaging time points. (B) LSFM images of *Tg(myl7:DsRed2-NLS)* larvae displaying ventricular cardiomyocytes at 48 hpi following transient treatment with 0.3% DMSO vehicle (left), 50 μM AT7519 (middle) or 3 μM FVP (right). (C) Number of ventricular cardiomyocytes at 24 hpi and 48 hpi following transient treatment with ≤0.3% DMSO vehicle, 50 μM AT7519 (left) or 3 μM FVP (right). Error bars represent s.d., *n*=29 larvae, experimental *n*=3. **P<0.01 (one-way ANOVA and Tukey post-hoc test for comparisons between DMSO vehicle or CDK9i treatment groups). (D) Surface-rendered LSFM image of a *Tg(myl7:GFP)* heart. Dashed line outlines the ventricular apex area that is subject to laser injury. (E) Surface-rendered LSFM image of a *Tg(myl7:GFP)* wound at the ventricular apex at 24 hpi and 48 hpi, following transient treatment with 0.1% DMSO vehicle (top) or 50 μM AT7519 (bottom). Arrowheads indicate cardiomyocyte protrusions adjacent to the wound (*myl7:GFP* negative). (F) Myocardial wound area (μm^2^) at 24 hpi and 48 hpi following transient treatment with 0.1% DMSO vehicle or 50 μM AT7519. Error bars represent s.d., *n*=27 larvae, experimental *n*=3. One-way ANOVA and Tukey post-hoc test performed for comparisons between treatment groups. (G) Myocardial wound closure (%) between 24 hpi and 48 hpi following transient treatment with 0.1% DMSO vehicle or 50 μM AT7519. Error bars represent s.d., *n*=27 larvae, experimental *n*=3. *P<0.05 (Mann–Whitney *U*-test for comparison between treatment groups). (H) Surface-rendered LSFM time-lapse-derived images of an injured *myl7:GFP* ventricle; hpi indicated. Arrowheads indicate myocardial wound (*myl7:GFP* negative). Asterisk indicates cardiomyocytes bridging across the myocardial wound (*myl7:GFP* negative). LSFM fluorescence images were acquired in 3D and maximum intensity projections (B) or 3D renders (D,E,H) are displayed. Scale bars: 50 μm (B,H); 20 μm (E). ns, non-significant.

We next tested whether the differing regulation of macrophage *tnf* polarisation following transient CDK9i treatment (Fig. 3) influenced cardiomyocyte numbers. Following transient AT7519 or FVP treatment (Fig. 5A), LSFM scans of *Tg(myl7:DsRed2-NLS)* larvae indicated no change in ventricular cardiomyocyte numbers at 24 hpi (Fig. 5C). At 48 hpi, however, ventricular cardiomyocyte numbers were significantly elevated following AT7519 treatment (321.6±31.7 versus 292.7±23.5) (Fig. 5B,C), indicating an increase in cardiomyocyte number expansion. At 48 hpi following FVP treatment no difference in cardiomyocyte numbers was observed (298.0±22.5 versus 298.8±25.8) (Fig. 5B,C). This AT7519-specific increase in cardiomyocyte numbers was injury specific as uninjured larvae displayed no change in ventricular cardiomyocyte numbers following the same treatment (Fig. S5).

### Transient AT7519 treatment accelerates structural myocardial regeneration following injury

We have previously shown that laser injury induces cardiomyocyte death locally at the ventricular apex (Kaveh et al., 2020). In order to determine whether the increase in cardiomyocyte numbers identified with transient AT7519 treatment is associated with improved structural myocardial regeneration, LSFM scans of cardiac-injured *Tg(myl7:GFP)* larvae were acquired and the ventricular wound area was quantified (Fig. 5D). At 24 hpi, the myocardial wound area was similar between DMSO vehicle-treated and AT7519-treated groups (109.2±84.8 μm^2^ versus 100.9±59.3 μm^2^) (Fig. 5E,F). At 48 hpi, both groups displayed a reduction in wound area, although the DMSO vehicle group trended towards a larger wound area compared with the AT7519 group (53.1±57.6 μm^2^ versus 29.9±30.2 μm^2^) (Fig. 5E,F). To determine the rate of wound regression in these groups, percentage myocardial wound closure between 24 hpi and 48 hpi was analysed. This indicated a clear increase in wound closure following AT7519 treatment compared with controls (69.6% versus 21.2%) (Fig. 5G), highlighting an acceleration in the rate of myocardial wound regression. To examine closely how the myocardial wound structurally regenerates, LSFM time-lapse images were acquired. Live heartbeat-synchronised time-lapse imaging revealed that wound-proximal cardiomyocytes protrude into, and subsequently bridge across, the injured myocardium to regenerate the damaged tissue (Movie 7, Fig. 5E,H).

### Macrophages are required for the improved cardiomyocyte regenerative response following AT7519 treatment

**Fig. 6.**
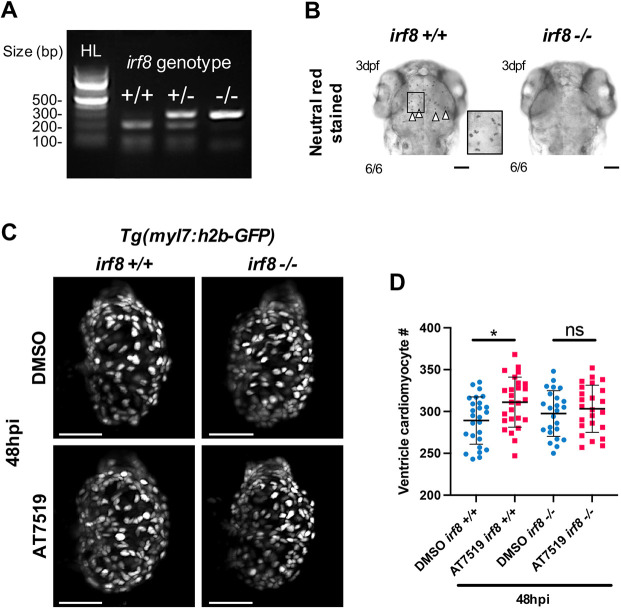
**Macrophages are required for enhanced cardiomyocyte number expansion following cardiac injury and transient AT7519 treatment.** (A) Restriction enzyme digest gel displaying *irf8* zebrafish genotypes. Hyperladder (HL) band and individual genotype bands (in order: *irf8^+/+^*, *irf8^+/−^* and *irf8^−/−^*) are indicated. (B) Brightfield images of 3 dpf larval heads stained with Neutral Red showing macrophages/microglia in the brain of *irf8^+/+^* larvae but not in *irf8^−/−^* larvae, with *irf8^−/−^* larvae devoid of all macrophages/microglia. Arrowheads indicate the presence of macrophages/microglia; inset shows a higher magnification of the boxed area. Scale bars: 500 μm. (C) LSFM images of *irf8^+/+^* and *irf8^−/−^ Tg(myl7:h2b-GFP)* larvae displaying ventricular cardiomyocytes at 48 hpi following transient treatment with 0.1% DMSO vehicle (top) or 50 μM AT7519 (bottom). Scale bars: 50 μm. (D) Number of ventricular cardiomyocytes at 48 hpi following transient treatment with 0.1% DMSO vehicle or 50 μM AT7519 in *irf8^+/+^* and *irf8^−/−^* larvae. Error bars represent s.d., *n*=25 larvae, experimental *n*=3. *P<0.05 (one-way ANOVA and Tukey post-hoc test for comparisons between DMSO vehicle or AT7519 treatment groups). ns, non-significant.

To determine whether macrophages are involved in the AT7519-associated increase in cardiomyocyte numbers following injury, we generated macrophage-null (*irf8* homozygous mutant) transgenic zebrafish to analyse cardiomyocyte nuclei numbers using LSFM. First, we incrossed heterozygous *irf8* mutants on a *Tg(myl7:h2b-GFP)* background and genotyped the offspring, selecting a population of wild type and homozygous *irf8* mutants (Fig. 6A). Neutral Red staining clearly demonstrated that *irf8^+/+^* larvae were marked with macrophages/microglia in the brain, whereas their *irf8^−/−^* counterparts completely lacked such staining (Shiau et al., 2015) and thus macrophages/microglia (Fig. 6B). Next, we cardiac injured macrophage-replete (*irf8^+/+^*) and macrophage-null (*irf8^−/−^*) larvae and subsequently administered the transient AT7519 treatment. Analysis of macrophage-replete (*irf8^+/+^*) larvae at 48 hpi showed an increase in cardiomyocyte numbers with AT7519 compared with DMSO vehicle, as established previously (Figs 6C,D and 5C). Macrophage-null (*irf8^−/−^*) larvae, however, did not display an injury-associated increase in cardiomyocyte numbers (Fig. 6C,D), suggesting that macrophages are required for the enhanced cardiomyocyte number expansion induced by transient AT7519 treatment.

## DISCUSSION

Resolving the inflammatory response is a promising therapeutic approach to promote tissue repair/regeneration following injury. CDK9 inhibitor compounds, currently deployed in clinical trials as anti-cancer treatments, can be applied experimentally to curtail early neutrophilic inflammation (Rossi et al., 2006; Lucas et al., 2014; Hoodless et al., 2016; Cartwright et al., 2019). This study is the first to examine the effect of CDK9 inhibitors (AT7519 and FVP) during the inflammatory and regenerative response following tissue injury *in vivo*. Using a larval zebrafish model of cardiac injury combined with heartbeat-synchronised imaging, we showed that AT7519 and FVP resolve neutrophilic inflammation at the injured heart via reverse migration, but differentially regulate macrophage polarisation and myocardial regeneration.

As previously shown in various models of injury and infection *in vivo* (Rossi et al., 2006; Loynes et al., 2010; Leitch et al., 2012; Lucas et al., 2014; Hoodless et al., 2016; Barth et al., 2020), we found that CDK9 inhibitors enhance the resolution of neutrophilic inflammation following heart injury in larval zebrafish. Numerous studies have demonstrated that CDK9 inhibitors induce neutrophil apoptosis via downregulation of Mcl1 (Moulding et al., 1998; Rossi, et al., 2006; Leitch et al., 2012; Wang et al., 2012; Lucas et al., 2014; Dorward et al., 2017). Here, we show that AT7519 and FVP promote the resolution of neutrophilic inflammation from the cardiac lesion via reverse migration (Fig. 1). Despite previously observing increased neutrophil apoptosis following tail fin transection with CDK9i treatment (Hoodless et al., 2016), we did not find any evidence of this at the injured heart. Reverse migration is the primary inflammatory-cell resolution mechanism in this model (Kaveh et al., 2020), most likely because of the size and sterility of the myocardial laser wound. An injury of such scale would release fewer chemoattractant signals, such as reactive oxygen species (e.g. hydrogen peroxide), cytokines (e.g. Il1β) and chemokines (e.g. Cxcr2/Cxcl8), responsible for regulating neutrophil wound retention (Yoo et al., 2010; Yan et al., 2014; Powell et al., 2017; Coombs et al., 2019; Isles et al., 2019). Expression of these inflammatory mediators could indeed be modulated in the presence of AT7519 or FVP, as documented in other studies (Santo et al., 2010; Yik et al., 2014). Consequently, this would alter the chemoattractant gradient, desensitising wound-swarming neutrophils and inducing their reverse migration. Similarly, other compounds that cause neutrophil apoptosis in mammalian systems have been shown to promote neutrophil reverse migration following tail fin wounding in larval zebrafish (Robertson et al., 2014). Further research is needed to understand better how CDK9 inhibitors regulate the aforementioned inflammatory mediators to induce immune cell reverse migration, particularly via chemokine signalling at sites of sterile injury (Isles et al., 2019; Coombs et al., 2019). Reverse migration may well be an important neutrophil resolution mechanism following cardiac injury in mammals, as shown following sterile liver injury (Wang et al., 2017). However, with live imaging proving extremely difficult in mammalian models of MI, it is not currently possible to visualise inflammatory cells non-invasively at high spatiotemporal resolution.

The majority of CDK9 inhibitors act by competitively inhibiting the ATP-binding domain, which is conserved between all CDKs (Kryštof et al., 2012). Consequently, long-term exposure to CDK9 inhibitor compounds can cause undesirable effects as a result of inhibition of other CDKs, many of which are cell-cycle regulators, such as CDK2 (De Azevedo et al., 1996; Wyatt et al., 2008). We showed that continuous AT7519 or FVP treatments result in developmental and injury-associated adverse effects, including reduced cardiomyocyte number expansion, cardiac function and macrophage wound retention, in addition to neutropenia (Fig. 2, Figs S1, S2). Continuous FVP treatment has previously been shown to inhibit cardiomyocyte proliferation in larval zebrafish (Matrone et al., 2015), suggesting that the same anti-proliferative effect could be occurring, although cardiomyocyte apoptosis may also contribute to the reduction in cardiomyocyte numbers. We postulated whether the adverse effects associated with continuous CDK9i treatment were due to non-specific binding. To test this, we developed a larval zebrafish CDK9 inhibitor selectivity assay using homozygous knockout *cdk9* mutants and heart rate as a surrogate measurement for overall health. The assay revealed that AT7519 and FVP accelerate the decline of heart rate in knockout mutants from 1 day post-treatment (Fig. 4). This reduction in heart rate coincides with the onset of adverse phenotypes (Fig. 2), indicating that from 1 day post-treatment both compounds were acting in a Cdk9-independent manner. However, the assay does not rule out the adverse effects being partially Cdk9 dependent, as vehicle-treated knockout mutants also displayed a decline in health, albeit more gradual. Of the two CDK9 inhibitors, FVP showed marked off-target effects in the selectivity assay (Fig. 4), a likely cause for the prominent adverse phenotypes observed (Fig. 2), which has also been reported *in vitro* (Garriga et al., 2010; Liu et al., 2012). Indeed, this larval zebrafish knockout screening approach could be applied to other druggable targets and used to identify uniquely selective inhibitors in a high-throughput manner and across short time scales (≤2 h) *in vivo*.

By limiting the CDK9i treatment period to a 2-h window, we were able to enhance the resolution of neutrophilic inflammation while avoiding all adverse effects. Using the transient treatment, wound macrophage accumulation was unaffected (Fig. 3), suggesting that prolonged neutrophil swarming is not required for macrophage recruitment/retention. Furthermore, we observed an unexpected difference between CDK9i treatments whereby AT7519, but not FVP, increased the polarisation of wound macrophages to a *tnf*^+^ phenotype (Fig. 3). The selectivity assay revealed that from 2 hpt (the duration of transient treatment), FVP exhibited significantly less Cdk9 selectivity compared with AT7519 (Fig. 4). Additionally, FVP has been shown to inhibit TNF activation and signalling in other models of inflammation (Takada and Aggarwal, 2004; Haque et al., 2011; Schmerwitz et al., 2011), whereas AT7519 does not disrupt TNF activity (Lucas et al., 2014). Overall, these findings suggest that FVP suppressed *tnf* upregulation in wound-associated macrophages. As our selectivity assay enables high-throughput assessment of individual animals across short time scales live *in vivo*, it is not suited for gene expression analysis. Therefore, how AT7519 and FVP differentially influence the expression of inflammatory response genes could be further investigated by RNA sequencing.

Cellular mechanisms regulating immune cell activity after wounding have been largely characterised in murine models and are not entirely recapitulated in zebrafish. For example, neutrophil apoptosis, subsequent macrophage efferocytosis and polarisation have not been reported following wounding in larval zebrafish (Starnes and Huttenlocher, 2012; Robertson et al., 2014; Loynes et al., 2018; Kaveh et al., 2020). Instead, the role of immune cells is more dynamic and closely coupled to molecular signalling (Loynes et al., 2018; Coombs et al., 2019; Tsarouchas et al., 2018; Sanz-Morejón et al., 2019). Larval zebrafish studies have demonstrated *tnf*^+^ macrophages to have pro-regenerative roles following tissue wounding (Nguyen-Chi et al., 2017; Tsarouchas et al., 2018; Gurevich et al., 2018; Cavone et al., 2021). Our data show that cardiac-injured larvae transiently treated with AT7519, but not FVP, exhibit enhanced cardiomyocyte number expansion at 2 days post-injury (Fig. 5), 1 day after the peak *tnf*^+^ macrophage response (Fig. 3). Importantly, we found macrophages to be required for the improved regenerative response following AT7519 treatment (Fig. 6). One molecular mechanism for this macrophage-dependent effect could be that *tnf*^+^ macrophages express/secrete mitogenic factors, such as Vegf, as is the case during muscle wounding angiogenesis (Gurevich et al., 2018). Similarly, Tnf itself could act as a mitogen via activation of histone genes in progenitor cells, as described during spinal cord regeneration (Cavone et al., 2021). Single-cell RNA sequencing of wound-dwelling macrophages has recently been performed in the spinal cord and skeletal musculature of larval zebrafish by Cavone et al. (2021) and Ratnayake et al. (2021), respectively. In both studies, the pro-regenerative macrophage subpopulation identified expresses traditional M1 and M2 markers and shares mitogenic factors, specifically Tnf and Hbegf (Cavone et al., 2021; Ratnayake et al., 2021). Given that *tnf*^+^ macrophages have pro-regenerative properties in larval zebrafish, *tnf* is likely one of many differentially regulated genes in macrophages that could be promoting cardiomyocyte regeneration in our model. Interestingly, in adult zebrafish *tnf^+^* macrophages promote scar deposition following cardiac injury (Bevan et al., 2020), suggesting a transition in *tnf^+^* macrophage function during zebrafish development.

Our data indicate that increased cardiomyocyte number expansion following transient AT7519 treatment correlates with accelerated myocardial wound closure, to the point of almost complete regeneration (Fig. 5). This was not, however, associated with enhanced cardiac function, which recovered rapidly in both AT7519-treated and control larvae. Cardiomyocyte proliferation is a prerequisite for cardiac regeneration in many animal models (Godwin et al., 2017; Chablais et al., 2011; Curado et al., 2007; Porrello et al., 2013), suggesting that cardiomyocyte proliferation, enhanced by macrophages in our model, could be driving myocardial wound closure. Furthermore, 4D LSFM imaging during myocardial regeneration revealed that wound-bordering cardiomyocytes protrude into and subsequently bridge across the wound, gradually sealing it (Fig. 5, Movie 7). Cardiomyocyte bridging has previously been reported following transplantation of neonatal rat cardiomyocytes to infarcted hearts *in vitro* (Sekine et al., 2006); however, to our knowledge, this is the first time such an event has been observed live in the beating heart. Extracellular matrix proteins, such as collagen, could form a scaffold to facilitate cardiomyocyte wound bridging, as similar mechanisms occur in the injured hearts of adult zebrafish (Simões et al., 2020). Future studies could include high-resolution live imaging and complementary sequencing experiments in larval zebrafish to unravel the cardiac/immune cell types and signalling molecules regulating cardiac regeneration.

As our findings are from a developing zebrafish model, it will be important to validate AT7519 treatment in an adult MI model that more closely mimics human disease. It will be particularly important to corroborate our findings by determining whether AT7519 polarises macrophages to a reparative phenotype and how this regulates cardiac fibrosis, cardiac function, scar resolution and angiogenesis. Furthermore, it will be necessary to identify whether AT7519 affects other immune cell types absent in our model, namely monocytes, B cells, T cells and eosinophils – all of which play important roles during myocardial injury and repair (Hofmann and Frantz, 2015; Toor et al., 2020). Nevertheless, we have shown that the timing, duration and selectivity of CDK9 inhibitor treatment is imperative when targeting the acute inflammatory response to promote tissue repair/regeneration. AT7519 treatment could be particularly effective in a clinical setting where MI is followed by prolonged coronary reperfusion injury, as there is a profound secondary influx of neutrophils (Vinten-Johansen, 2004; Niccoli et al., 2009; Mangold et al., 2015). This can occur following percutaneous coronary intervention (PCI), a standard clinical procedure for opening an acutely occluded coronary artery following MI. Thus, AT7519 could be administered at the time of PCI to resolve locally recruited neutrophils and promote downstream mechanisms that positively modulate myocardial repair.

In summary, we have shown that AT7519 and FVP resolve neutrophil infiltration by inducing reverse migration from the cardiac injury site. However, AT7519, unlike FVP, showed promise as a selective CDK9 inhibitor by augmenting macrophage polarisation and promoting cardiomyocyte regeneration. As such, future research should establish whether selective CDK9 inhibitors, such as AT7519, have analogous reparative effects on macrophage polarisation and infarct healing in adult models of MI associated with neutrophilic inflammation. This could ultimately reveal the clinical potential of selective CDK9 inhibition as an immunomodulatory therapy for MI.

## MATERIALS AND METHODS

### Zebrafish husbandry and lines used

Zebrafish husbandry and maintenance was conducted as per standard operating procedures. This was in accordance with the Animals (Scientific Procedures) Act, 1986 and approved by The University of Edinburgh Animal Welfare and Ethical Review Board in a UK Home Office-approved establishment. All experiments were performed on staged animals aged between 3 dpf and 5 dpf (Kimmel et al., 1995). The following zebrafish lines were used: *Tg(myl7:eGFP)^twu^*^26^ (Huang et al., 2003), *Tg(mpx:mCherry)^uwm^*^7^ (Yoo et al., 2010), *Tg(mpeg1:mCherry)^gl^*^23^ (Ellett et al., 2011), *Tg(myl7:DsRed2-NLS)^f^*^2^ (Rottbauer et al., 2002), *Tg(TNFa:eGFP)^sa43296^* (Nguyen-Chi et al., 2015), *Tg(myl7:h2b-GFP)^zf^*^52^ (Mickoleit et al., 2014), *cdk9^ed9^* mutant (Hoodless et al., 2016) and *irf8^st59/st95^* mutant (Shiau et al., 2015). Adults were bred to yield the desired combinations of transgenes in embryos. Embryos were treated with 0.003% phenylthiourea (Fisher Scientific) dissolved in conditioned water at 7 h post-fertilisation (hpf) to prevent pigment formation and enhance image clarity (Karlsson et al., 2001). Embryos and larvae were housed at 28.5°C in conditioned water and imaged at room temperature (23°C) using epifluorescence or light-sheet fluorescence microscopy (see below for details). When necessary, larvae were periodically anaesthetised using 40 μg/ml tricaine methanesulphonate (Sigma-Aldrich) in conditioned water.

### Cardiac injury

The hearts of 72 hpf larval zebrafish were injured precisely using a Zeiss Photo Activated Laser Microdissection (PALM) system, as previously described (Taylor et al., 2019; Kaveh et al., 2020). Individual anaesthetised larvae were pipetted onto a glass slide in 20 μl conditioned water containing tricaine methanesulphonate and laterally oriented so that the head is pointing leftward. Larvae were positioned adjacent to each other and found on the slide using the automated PALM controls. The laser was focussed specifically on the ventricular apex and subsequently fired through a 20× objective. Hearts were typically laser-pulsed three times along the ventricular apex (Fig. S1) until ventricular contractility had diminished, the apex had shrunk, and the myocardial wall had become swollen. Cardiac-ruptured larvae that displayed pericardial bleeding following laser injury were appropriately discarded. Larvae were deemed injured if they displayed a loss of fluorescent myocardial transgenic signal and/or a robust immune cell recruitment response at the cardiac injury site. Uninjured (control) larvae were treated in the same manner up to the point of laser injury, when they were separated and maintained in the same environmental conditions as injured fish.

### Pharmacological CDK9 inhibitor treatment

Larvae were incubated in AT7519 (Astex Pharmaceuticals), FVP or DMSO vehicle (both Sigma-Aldrich) dissolved in phenylthiourea-treated conditioned water at the following concentrations: 1 μM or 50 μM AT7519, 1 μM or 3 μM flavopiridol, and/or 0.1% or 0.3% DMSO vehicle from 72 hpf or 4 hpi, depending on the experiment as indicated. For continuous treatments, larvae were incubated in drug or vehicle from 4 hpi until 24 hpi or 48 hpi. For transient treatments, larvae were incubated in drug or vehicle from 4 hpi until 6 hpi, at which point they were transferred to fresh conditioned water. For serial time-point experiments, individual larvae were incubated in separate wells in a 48-well plate containing 500 μl of CDK9 inhibitor or DMSO vehicle in conditioned water. During these experiments, larvae were briefly removed for imaging at 6 hpi, 24 hpi or 48 hpi. For LSFM time-lapse experiments, individual anaesthetised larvae were embedded in 1% low melting point agarose (Thermo Fisher) in conditioned water containing 50 μM AT7519, 3 μM flavopiridol or DMSO vehicle within FEP tubes (Adtech Polymer Engineering). During LSFM time-lapse imaging, larvae were continually anaesthetised using tricaine methanesulphonate-conditioned water containing 50 μM AT7519, 3 μM flavopiridol or DMSO vehicle, as appropriate for up to 24 h.

### Epifluorescence microscopy

A Leica M205 FA stereomicroscope with standard GFP and mCherry filters was used for serial time-point imaging experiments. To visualise immune cells or record cardiac function, larvae were anaesthetised and mounted laterally on a glass slide in 50 μl conditioned water. Immune cell numbers on the ventricle were quantified by counting neutrophils or macrophages moving synchronously with the beating heart, as performed previously (Kaveh et al., 2020). Cardiac images were acquired using a 16× objective and whole-body images were acquired using a 2.5× objective.

### Heartbeat-synchronised light-sheet fluorescence microscopy (LSFM)

Optically gated (heartbeat-synchronised) LSFM imaging methods have been thoroughly described and published by our group (Taylor et al., 2019; Kaveh et al., 2020). Briefly, bespoke synchronisation software coupled with LSFM allows real-time 3D fluorescence imaging of the beating heart every time the heart returns to a desired target phase of the cardiac cycle, while minimising phototoxicity and photobleaching (Taylor et al., 2019). As a result, the beating heart appears computationally ‘frozen’, allowing live examination of immune cell responses and cardiomyocyte regeneration following injury *in vivo*. Each heart stack has a *z*-plane spacing of 1 μm. For time-lapse imaging, the entire heart is scanned in 3D every 3 min for up to 24 h.

### CDK9 inhibitor selectivity assay

Knockout *cdk9* mutant zebrafish were previously generated and characterised by our group (Hoodless et al., 2016). As homozygous *cdk9* mutants are not viable at adulthood, adult heterozygous *cdk9* mutants were identified by genotyping (Hoodless et al., 2016) and incrossed, yielding a Mendelian mix of wild-type (25%), heterozygous (50%) and homozygous mutant (25%) zebrafish embryos. Homozygous *cdk9* mutants are phenotypically distinguishable during larval stages (Fig. 4A), which was confirmed by genotyping (Fig. 4B). At 72 hpf, *cdk9* homozygous mutant larvae were phenotypically selected and treated with AT7519, FVP or DMSO vehicle, at the indicated doses. Following this, heart rate (beats/minute) was measured per larva by manually counting heartbeats per 12 sec using a brightfield stereomicroscope and multiplying by five. This was performed between groups at 2 hpt, 6 hpt, 12 hpt, 24 hpt and 48 hpt as a proxy for overall health, allowing individual larvae to be assessed in real time. Larvae that did not display any heartbeat were regarded as dead.

### Preparing homozygous *irf8* (macrophage-null) mutants

Adult homozygous *irf8* mutants were outcrossed to *Tg(myl7:h2b-GFP)* fish and the transgenic offspring were raised to adulthood. Adult heterozygous *irf8* mutants were further incrossed and the offspring raised to adulthood. Adult zebrafish arising from the heterozygous *irf8* mutant incrosses were genotyped to identify wild-type, heterozygous or homozygous mutant *irf8* alleles. First, adult fish were anaesthetised in 40 μg/ml tricaine methanesulphonate and a section of one tail fin lobe was resected using a sterile scalpel. Tail fin clips were digested to extract DNA using 10 mg/ml of proteinase K, by incubating at 67°C for 1 h. The digest was ended with a 95°C incubation for 15 min. The *irf8* allele was amplified from the extracted DNA by PCR using forward (ACATAAGGCGTAGAGATTGGACG) and reverse (GAAACATAGTGCGGTCCTCATCC) primers and REDTaq ReadyMix PCR Reaction Mix (Sigma-Aldrich). The PCR product was then digested for 1 h at 37°C using the restriction enzyme AvaI (New England Bioscience) and the product run on a 2% agarose gel to visualise digested DNA fragments. In wild-type fish, the AvaI cut site is intact, thus the PCR product is digested to give two bands approximately 200 bp and 100 bp in size. In heterozygous mutant fish, AvaI partially digests the PCR product. In homozygous mutant fish, the AvaI cut site is not present, so the 286 bp PCR product is not digested. Confirmed wild-type and homozygous *irf8* mutant adults were separated for experimental incrossing.

### Neutral Red staining

Wild-type or homozygous *irf8* mutant larvae at 3 dpf were incubated in 5 μg/ml Neutral Red (Thermo Fisher Scientific) in conditioned water for 1 h in the dark at 28.5°C. Larvae were then washed twice in conditioned water, anaesthetised using 40 μg/ml tricaine methanesulphonate and imaged by brightfield microscopy on a Leica M205 FA stereomicroscope.

### Image analysis

Unless otherwise stated, all images were prepared, processed and analysed using ImageJ (Fiji) software (National Institutes of Health).

### Temporal colour code

LSFM-acquired *z*-stacks of neutrophil or macrophage migration on injured hearts were processed as maximum intensity projections and temporally overlaid across the indicated time points. The ‘Temporal-Color Code’ Fiji tool was applied to the hyperstack such that each overlaid time point is a different hue, producing a single image that summarises immune cell migration on the injured heart.

### Ventricular cardiomyocyte number

Individual LSFM-acquired *z*-stacks of *Tg(myl7:DsRed2-NLS)* or *Tg(myl7:h2b-GFP)* hearts were used to quantify the number of cardiomyocyte nuclei using the ‘TrackMate’ Fiji plugin. The following segmentation parameters were initially applied to each *z*-stack: ‘LoG detector’, ‘Median filter’ and ‘Sub-pixel localisation’ selected; ‘Estimated blob diameter’=6 μm and ‘Threshold’=1. The threshold value was optimised per experiment until all cardiomyocyte nuclei were included. Atrial cardiomyocytes were subtracted from the total by *x* coordinate filtering to give a final ventricular cardiomyocyte count.

### Ventricular ejection fraction

The hearts of *Tg(myl7:GFP)* larvae were imaged in real time at 30 frames per second using epifluorescence microscopy to capture points in the cardiac cycle when the ventricle was in diastole and systole. Ventricular area in diastole and systole was measured manually and ventricular ejection fraction (by area) was calculated using the formula: 100×[(diastolic area−systolic area)/diastolic area] (Matrone et al., 2013).

### Whole-body immune cell number

Whole-body epifluorescence images of *Tg(mpx:mCherry)* or *Tg(mpeg1:mCherry)* larvae were used to estimate global immune cell numbers in a semi-automated manner using a Fiji macro. Briefly, individual images were thresholded using the ‘Yen’ technique, converted to binary and whole-body immune cell thresholded area was quantified. The area of three thresholded immune cells was measured at random. Whole-body immune cell threshold area was divided by the average immune cell area to estimate global immune cell numbers per larva.

### Ventricular *tnf^+^* macrophage number

Individual LSFM-acquired *z*-stacks of *Tg(mpeg1:mcherry;TNFa:GFP)* hearts were processed as maximum intensity projections to visualise macrophages and *tnf* expression throughout the heart. The number of ventricular macrophages expressing *tnf* (above that of background levels) were counted. *Tg(mpeg1:mcherry)* larvae were used as a measure of background fluorescence and a negative control in this context.

### Myocardial wound area

Individual LSFM-acquired *z*-stacks of *Tg(myl7:GFP)* injured hearts were 3D rendered using Imaris software (Bitplane) based on absolute intensity, suggested segmentation and rendering parameters. Rendered hearts were saved as separate images and imported into Fiji. The *myl7:GFP*-negative area at the ventricular apex (visualised as a render-free hole in the myocardium) was manually traced around and quantified to give myocardial wound area (μm^2^) (Fig. 5D,E).

### Randomisation and blinding

At the start of each experiment, larvae were screened for the relevant fluorescent signals and then randomly allocated to different experimental groups. All analysis was performed blind to treatment groups.

### Statistical analysis

Graphs were curated and statistical analysis was performed using GraphPad Prism 9 software. The normal distribution of quantitative data was confirmed using the Shapiro–Wilk test and subsequently analysed using parametric or non-parametric tests, as appropriate. If normally distributed, data were analysed by one-way ANOVA or two-way ANOVA followed by a multiple comparison post-hoc test. If not normally distributed, data were analysed using the Mann–Whitney *U*-test. Error bars indicate standard error of the mean (s.e.m.) or standard deviation (s.d.). All statistical tests, *P*-values and *n* numbers are stated in figure legends.

## Supplementary Material

Supplementary information

Reviewer comments
