# Supplementary material for: Selective Cdk9 inhibition resolves neutrophilic inflammation and enhances cardiac regeneration in larval zebrafish
Source: Development. 2021 Oct 26;149(8):dev199636. doi: 10.1242/dev.199636 (PMC8601713; doi:10.1242/dev.199636)
Supplement: Supplementary information [file develop-149-199636-s1.pdf]

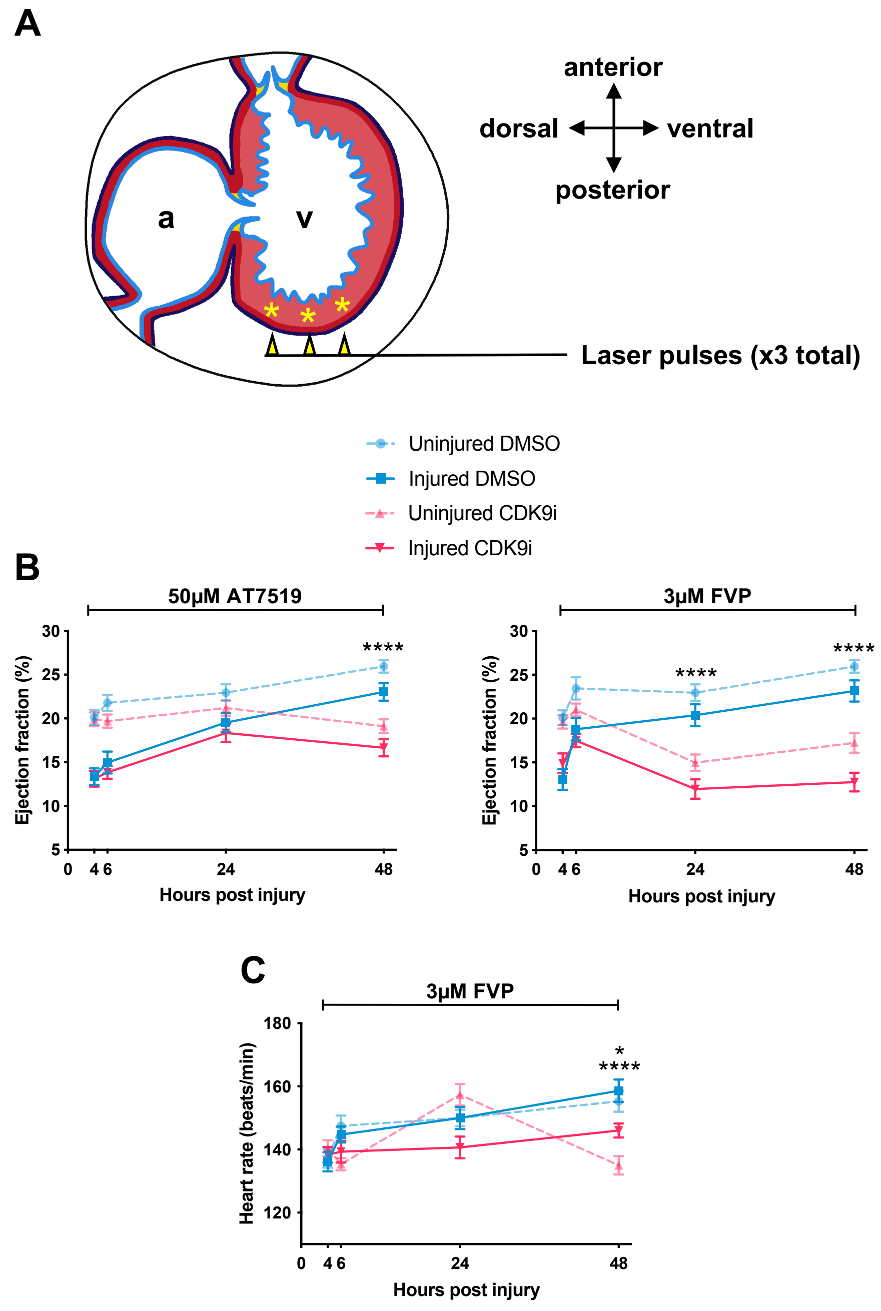

Fig. S1. Continuous CDK9i treatment reduces heart function. (A) Schematic illustrating the method of cardiac injury by laser pulsing along the ventricular apex of 3 dpf zebrafish larval hearts. Asterisks indicate the three sites of laser injury. Atrium = a and ventricle = v. **(B)** Ventricular ejection fraction (%) at 4 hpi, 6 hpi, 24 hpi and 48 hpi with ≤0.3% DMSO vehicle, 50µM AT7519 (left graph) or 3µM FVP (right graph). Error bars = SEM, *n* = 18 larvae, experimental *n* = 3. Two-way ANOVA and Bonferroni *post hoc* test performed for comparisons between DMSO vehicle or CDK9i treatment groups, where **** *p < 0.0001*. **(C)** Heart rate (beats/minute) at 4 hpi, 6 hpi, 24 hpi and 48 hpi with 0.3% DMSO vehicle or 3µM FVP. Error bars = SEM, *n* = 15 larvae, experimental *n* = 3. Two-way ANOVA and Bonferroni *post hoc* test performed for comparisons between DMSO vehicle or FVP treatment groups, where ** *p < 0.01* and **** *p < 0.0001.*

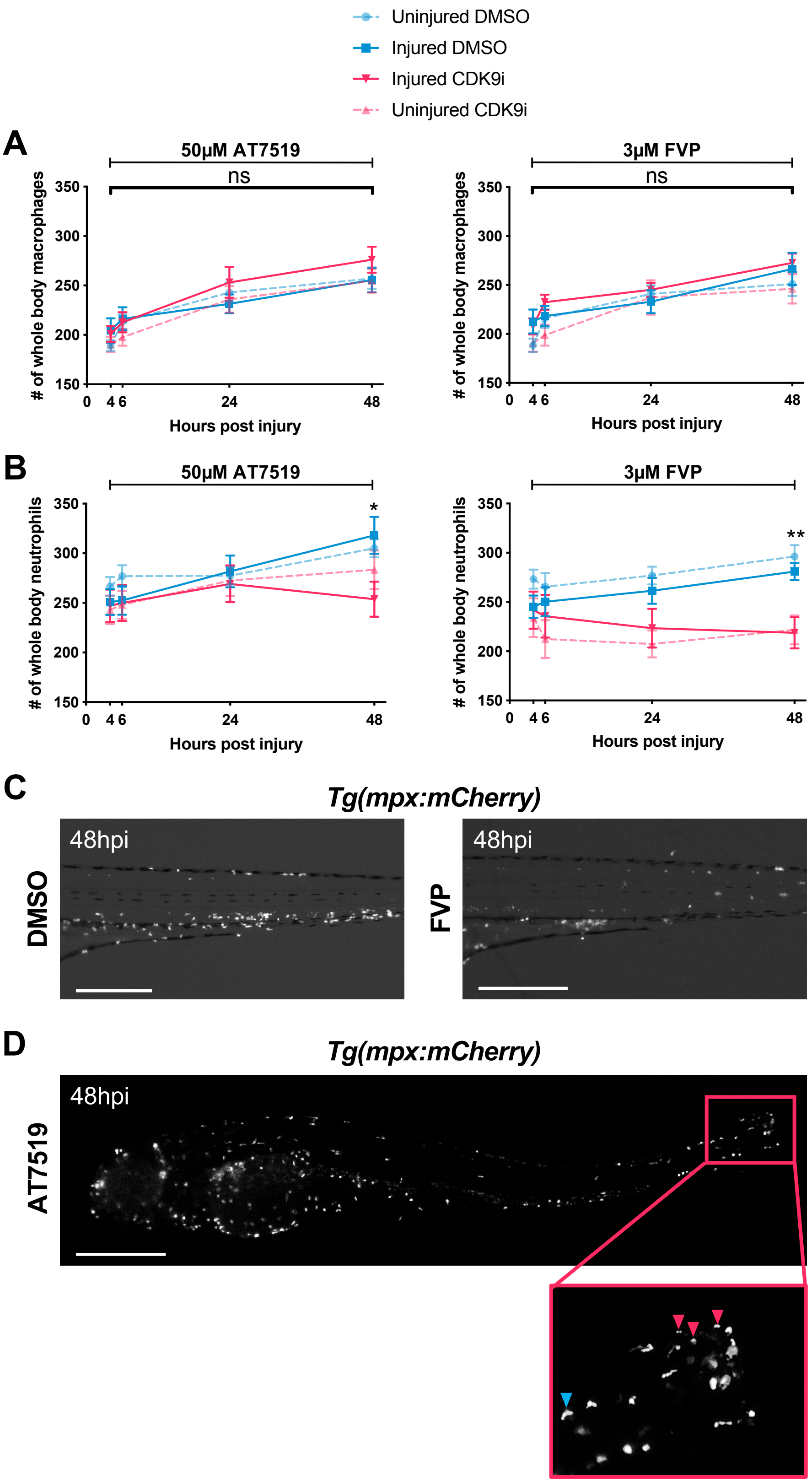

Development • Supplementary information

Fig. S2. Continuous CDK9i treatment does not affect global macrophage numbers but causes neutropenia. (A) Number of whole-body macrophages at 4 hpi, 6 hpi, 24 hpi and 48 hpi, with ≤0.3% DMSO vehicle, 50μM AT7519 (left graph) or 3μM FVP (right graph). Error bars = SEM, n = 18 larvae, experimental n = 3. Two-way ANOVA and Bonferroni *post hoc* test performed for comparisons between DMSO vehicle or CDK9i treatment groups. Ns, non-significant. **(B)** Number of whole-body neutrophils at 4 hpi, 6 hpi, 24 hpi and 48 hpi with ≤0.3%, DMSO vehicle, 50μM AT7519 (left graph) or 3μM FVP (right graph). Error bars = SEM, n = 16 larvae, experimental n = 3. Two-way ANOVA and Bonferroni *post hoc* test performed for comparisons between DMSO vehicle or CDK9i treatment groups, where * $p < 0.05$ and ** $p < 0.01$. **(C)** Epifluorescence image of *Tg(mpx:mCherry)* neutrophils within the caudal hematopoietic region at 48 hpi following treatment with 0.3% DMSO vehicle (left) or 3μM FVP (right). Scale bars = 300 μm. **(D)** Epifluorescence image of *Tg(mpx:mCherry)* neutrophils within a whole larva at 48 hpi following treatment with 50μM AT7519. Scale bar = 500 μm. Red panel indicates area of magnified view below whole larva image. Within magnified view: blue arrowhead indicates a normal migrating neutrophil and red arrowheads indicate condensed and rounded neutrophils.

Development • Supplementary information

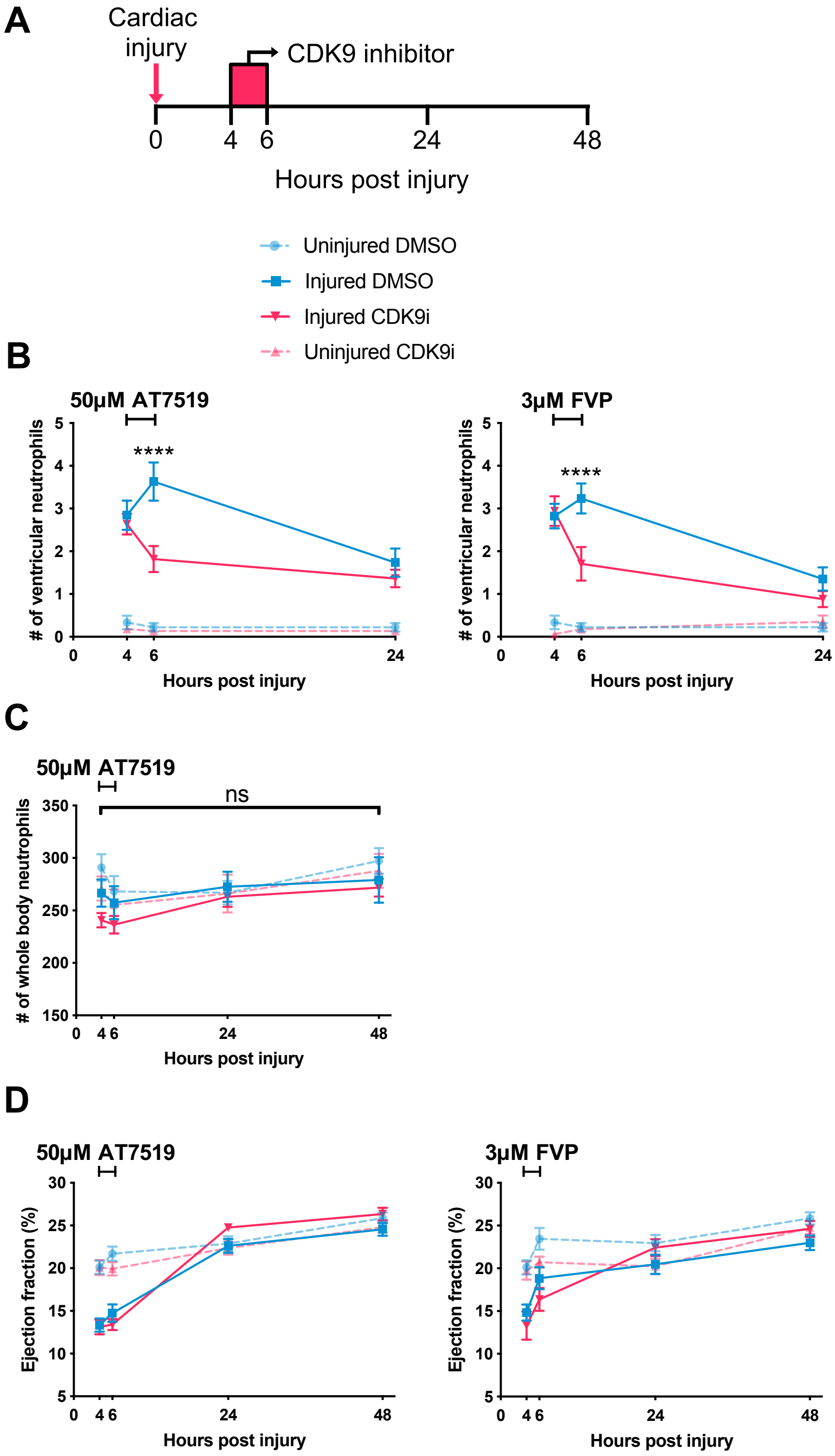

Development • Supplementary information

Fig. S3. Transient CDK9i treatment resolves neutrophilic inflammation without causing neutropenia or impairing cardiac contractility. (A) Experimental timeline indicating cardiac injury, transient CDK9i treatment and imaging timepoints. **(B)** Number of ventricular neutrophils at 4 hpi, 6 hpi and 24 hpi with 50μM AT7519 (left graph) or 3μM FVP (right graph) transient (pulsed) treatment. Error bars = SEM, *n* = 18 larvae, experimental *n* = 3. Two-way ANOVA and Bonferroni *post hoc* test performed for comparisons between cardiac-injured DMSO vehicle or CDK9i treatment groups where **** $p < 0.0001$. **(C)** Number of whole-body neutrophils at 4 hpi, 6 hpi, 24 hpi and 48 hpi following transient treatment with 0.1% DMSO vehicle or 50μM AT7519. Error bars = SEM, *n* = 17 larvae, experimental *n* = 3. Two-way ANOVA and Bonferroni *post hoc* test performed for comparisons between treatment groups. Ns, non-significant. **(D)** Ventricular ejection fraction (%) at 4 hpi, 6 hpi, 24 hpi and 48 hpi following transient treatment with ≤0.3% DMSO vehicle, 50μM AT7519 (left graph) or 3μM FVP (right graph). Error bars = SEM, *n* = 18 larvae, experimental *n* = 3. Two-way ANOVA and Bonferroni *post hoc* test performed for comparisons between DMSO vehicle or CDK9i treatment groups. No statistical differences were observed between injured DMSO vehicle and CDK9i treatment groups across all timepoints.

Development • Supplementary information

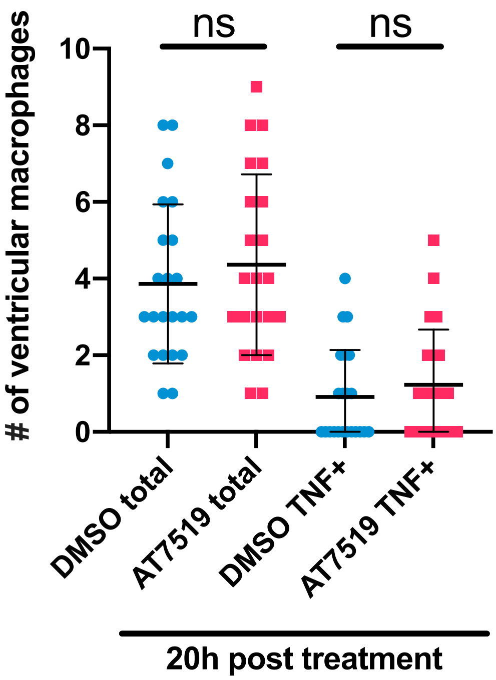

Fig. S4. Transient AT7519 treatment does not polarise cardiac macrophages to a *tnf*⁺ phenotype in uninjured larvae. Number of ventricular macrophages (total and *tnf*⁺) at 20 hpt following transient treatment with 0.1% DMSO vehicle or 50µM AT7519 in uninjured larvae. Error bars = SD, *n* = 23 larvae, experimental *n* = 3. One-way ANOVA and Tukey *post hoc* test performed for comparisons between treatment groups. Ns, non-significant.

Development • Supplementary information

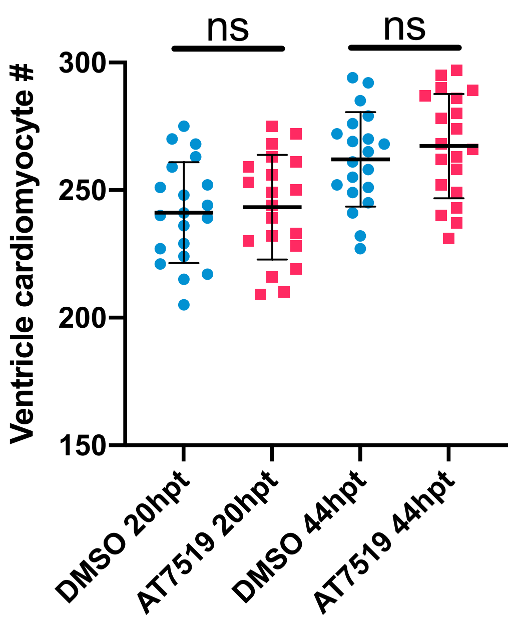

Fig. S5. Transient AT7519 treatment does not enhance cardiomyocyte number expansion in uninjured larvae. Number of ventricular cardiomyocytes in uninjured larvae at 24 hpt and 48 hpt following transient treatment with 0.1% DMSO vehicle or 50μM AT7519. Error bars = SD, *n* = 21 larvae, experimental *n* = 3. One-way ANOVA and Tukey *post hoc* test performed for comparisons between treatment groups. Ns, non-significant.

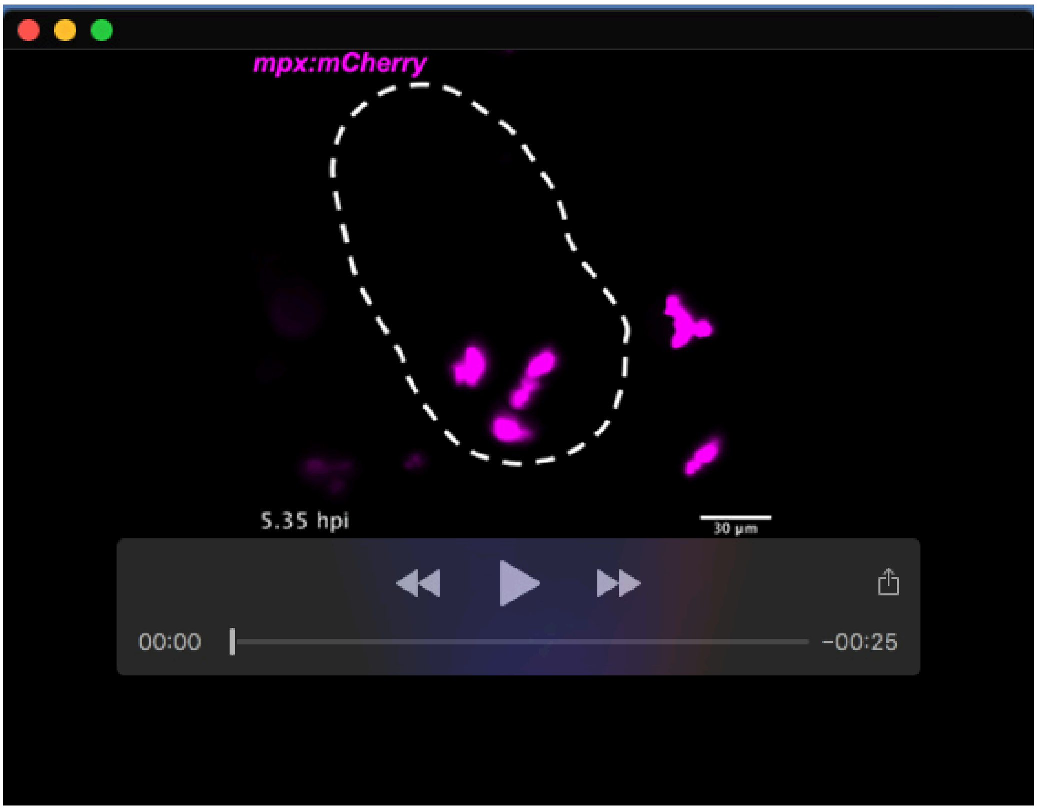

Movie 1. Neutrophil infiltration following cardiac injury with DMSO vehicle
LSFM heartbeat-synchronised timelapse of a *Tg(mpx:mCherry)* larva treated with DMSO vehicle showing neutrophils infiltrating the cardiac injury site between 4 and 6 hpi. 3D images are displayed as maximum intensity projections. Dotted line indicates outline of ventricle.

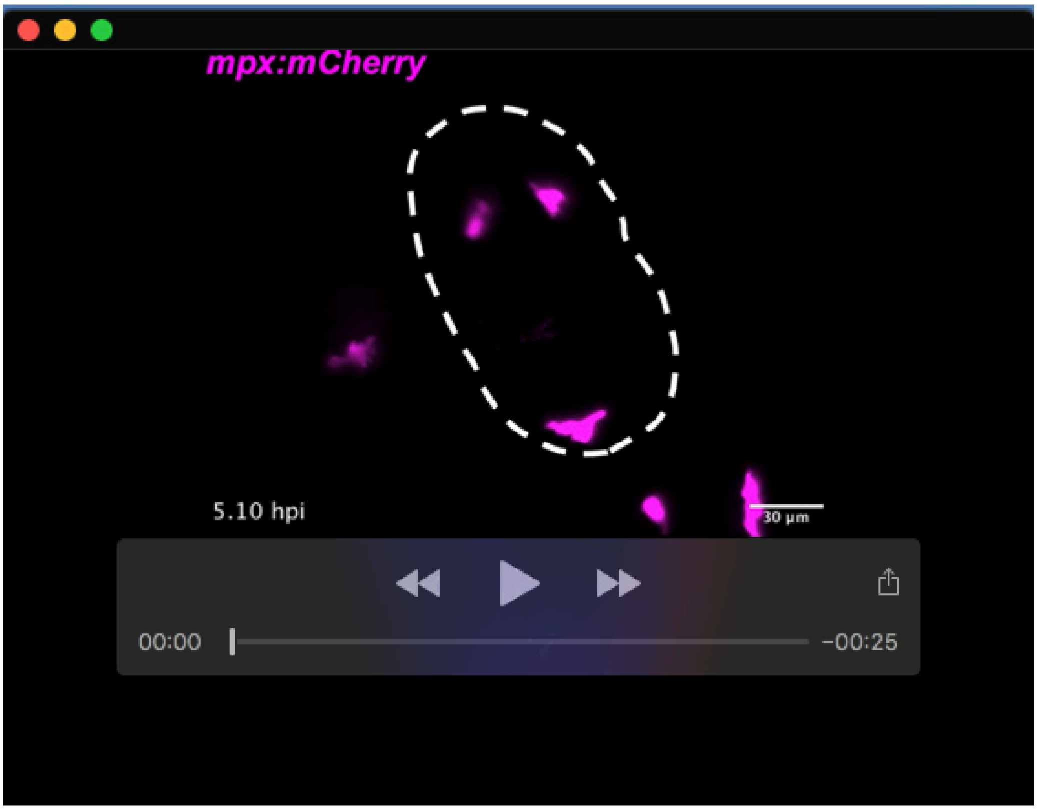

Movie 2. Neutrophil reverse migration following cardiac injury with AT7519
LSFM heartbeat-synchronised timelapse of a *Tg(mpx:mCherry)* larva treated with AT7519 showing wound-recruited neutrophils undergoing reverse migration from the injured heart between 4 and 6 hpi. 3D images are displayed as maximum intensity projections. Dotted line indicates outline of ventricle.

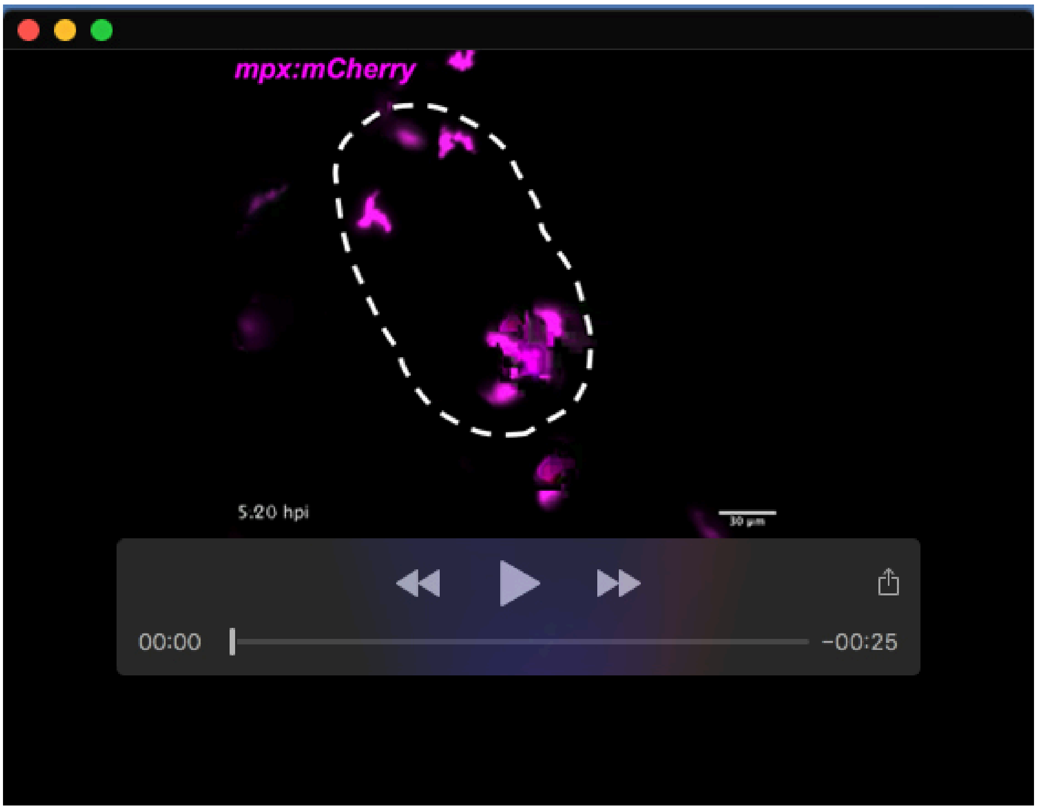

Movie 3. Neutrophil reverse migration following cardiac injury with Flavopiridol
LSFM heartbeat-synchronised timelapse of a *Tg(mpx:mCherry)* larva treated with Flavopiridol showing wound-recruited neutrophils undergoing reverse migration from the injured heart between 4 and 6 hpi. 3D images are displayed as maximum intensity projections. Dotted line indicates outline of ventricle.

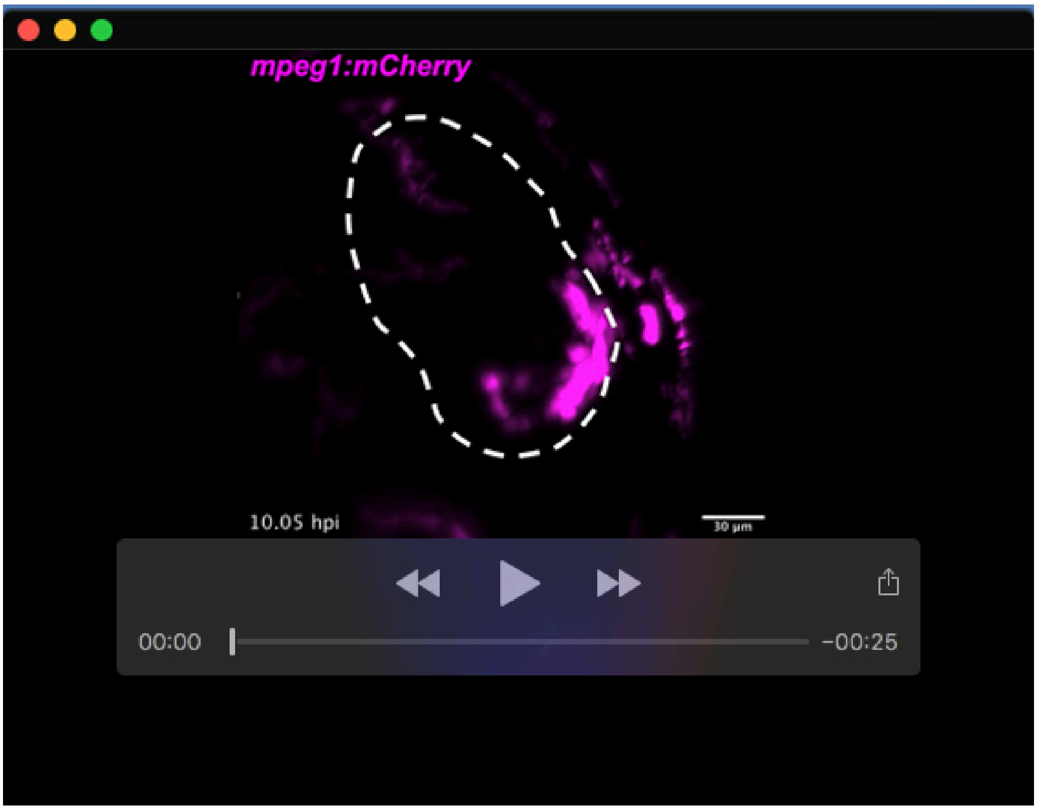

Movie 4. Macrophage wound retention following cardiac injury with DMSO vehicle LSFM heartbeat-synchronised timelapse of a *Tg(mpeg1:mCherry)* larva treated with DMSO vehicle showing wound-recruited macrophages being retained on the injured heart between 4 and 20 hpi. 3D images are displayed as maximum intensity projections. Dotted line indicates outline of ventricle.

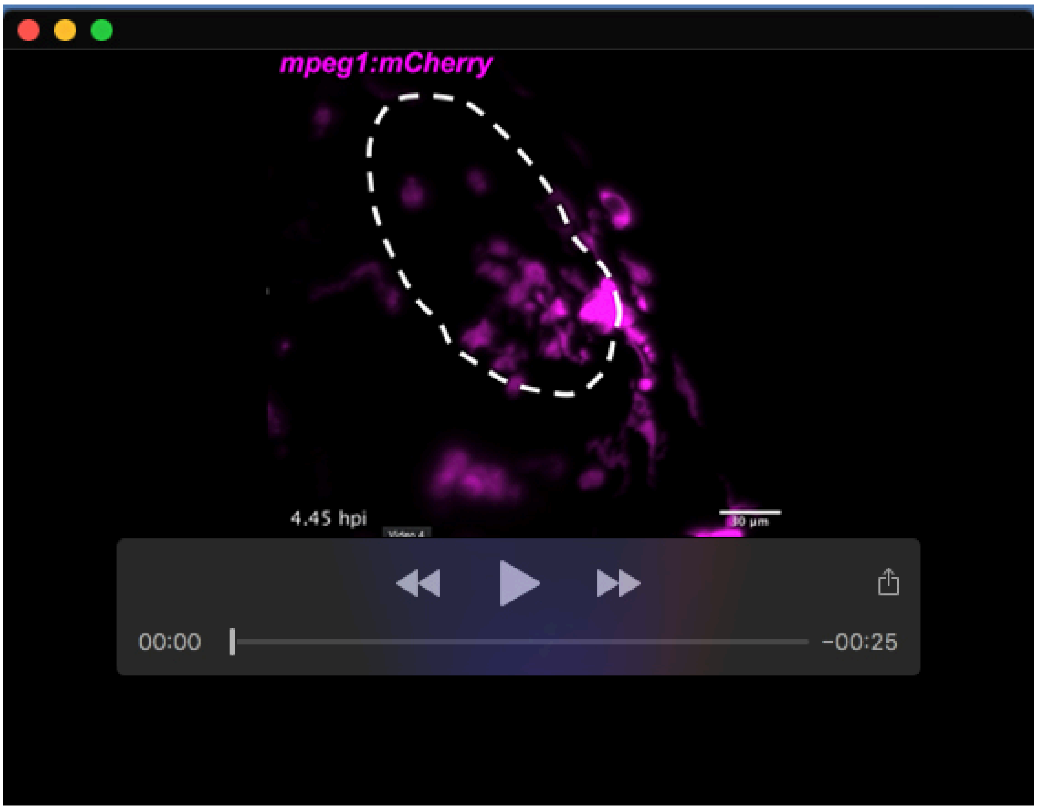

Movie 5. Macrophage reverse migration following cardiac injury with Flavopiridol LSFM heartbeat-synchronised timelapse of a *Tg(mpeg1:mCherry)* larva treated with Flavopiridol showing wound-recruited macrophages undergoing reverse migration from the injured heart between 4 and 21 hpi. 3D images are displayed as maximum intensity projections. Dotted line indicates outline of ventricle.

Development • Supplementary information

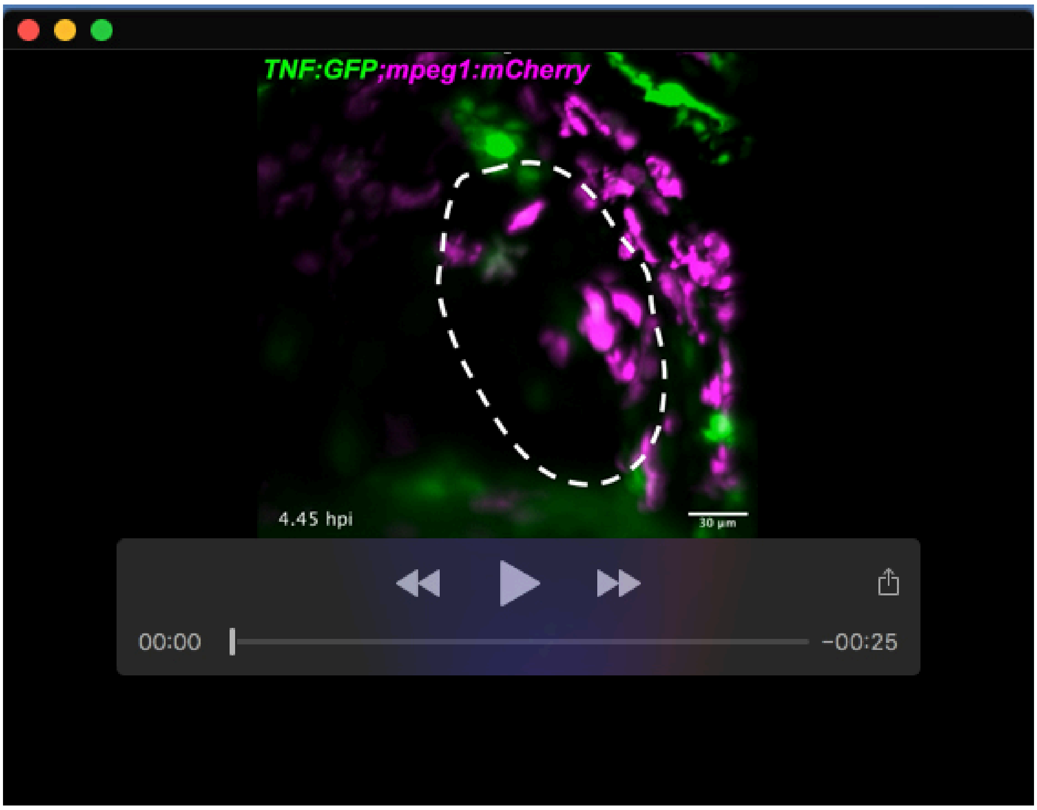

Movie 6. Wound-associated macrophages upregulate *tnf* expression following cardiac injury

LSFM heartbeat-synchronised timelapse of a *Tg(mpeg1:mCherry;TNFa:GFP)* larva showing wound-adjacent macrophages upregulating *tnf* expression at the cardiac injury site between 4 and 15 hpi. 3D images are displayed as maximum intensity projections. Dotted line indicates outline of ventricle.

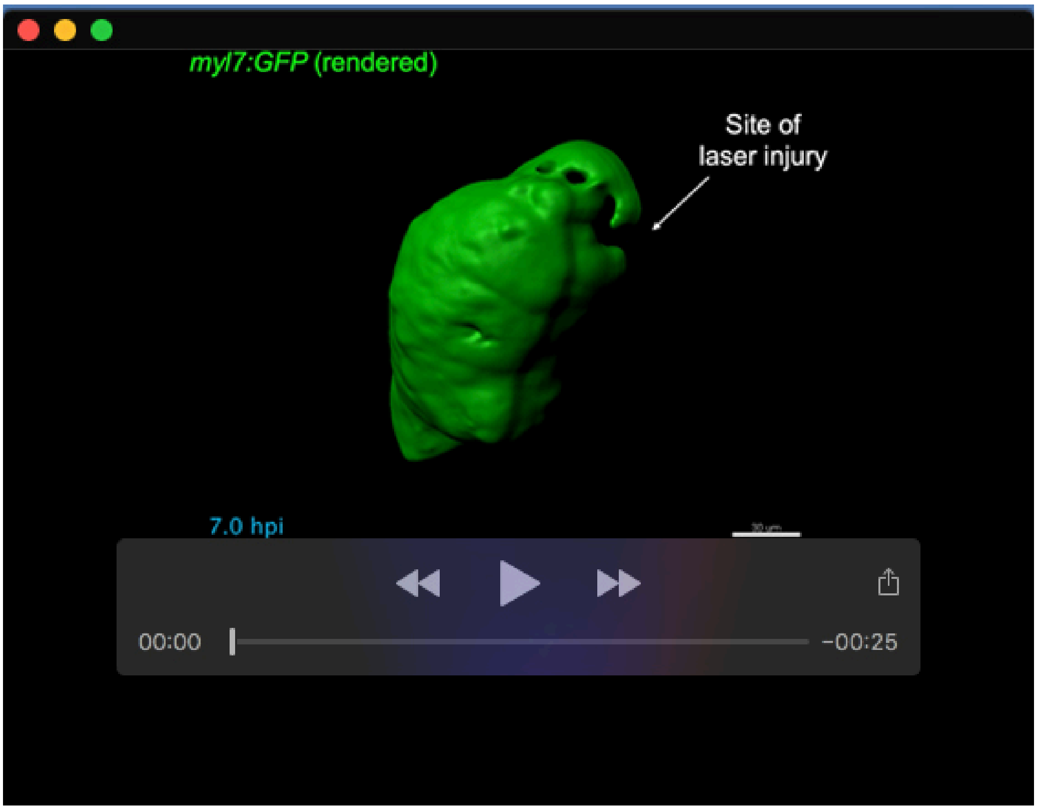

Movie 7. Myocardial regeneration via cardiomyocyte protrusion and bridging following cardiac injury

LSFM heartbeat-synchronised timelapse of a *Tg(myl7:GFP)* larva showing myocardial regeneration occurring via cardiomyocyte protrusion and bridging between 6 and 17 hpi. 3D images were surface rendered.
