# Supplementary material for: Selective Cdk9 inhibition resolves neutrophilic inflammation and enhances cardiac regeneration in larval zebrafish
Source: Development. 2021 Oct 26;149(8):dev199636. doi: 10.1242/dev.199636 (PMC8601713; doi:10.1242/dev.199636)
Supplement: Reviewer comments [file develop-149-199636_review_history.pdf]

Selective CDK9 inhibition resolves neutrophilic inflammation and enhances cardiac regeneration in larval zebrafish

Aryan Kaveh, Finnius A Bruton, Magdalena E M Oremek, Carl S Tucker, Jonathan M Taylor, John J Mullins, Adriano G Rossi and Martin A Denvir
DOI: 10.1242/dev.199636

Editor: Paul Martin

Review timeline
Original submission: 26 March 2021
Editorial decision: 24 May 2021
First revision received: 28 July 2021
Accepted: 20 August 2021

Original submission

First decision letter

MS ID#: DEVELOP/2021/199636

MS TITLE: Selective CDK9 inhibition resolves neutrophilic inflammation and enhances cardiac regeneration in larval zebrafish

AUTHORS: Aryan Kaveh, Finnius A Bruton, Magdalena E M Oremek, Carl S Tucker, Jonathan M Taylor, John J Mullins, Adriano G Rossi, and Martin A Denvir

I have now received all the referees' reports on the above manuscript, and have reached a decision. The referees' comments are appended below, or you can access them online: please go to BenchPress and click on the 'Manuscripts with Decisions' queue in the Author Area.

As you will see, while all three referees are excited by the potential clinical implications of your study, all three had substantial quibbles and none are very enthusiastic about it being suitable for publication without substantial additional work, for example some additional verification studies in an adult cardiac repair model. I would be very happy to see a revised version of your paper if you feel you could satisfy the majority of their concerns - particularly those of reviewers 2 and 3 - in a reasonable time frame.Your revised paper will be re-reviewed by one or more of the original referees, and acceptance of your manuscript will depend on your addressing satisfactorily the reviewers' major concerns. Please also note that Development will normally permit only one round of major revision.

We are aware that you may be experiencing disruption to the normal running of your lab that make experimental revisions challenging. If it would be helpful, we encourage you to contact us to discuss your revision in greater detail. Please send us a point-by-point response indicating where you are able to address concerns raised (either experimentally or by changes to the text) and where you will not be able to do so within the normal timeframe of a revision. We will then provide further guidance. Please also note that we are happy to extend revision timeframes as necessary.

Please attend to all of the reviewers' comments and ensure that you clearly highlight all changes made in the revised manuscript. Please avoid using 'Tracked changes' in Word files as these are lost in PDF conversion. I should be grateful if you would also provide a point-by-point response detailing

© 2021. Published by The Company of Biologists under the terms of the Creative Commons Attribution License (https://creativecommons.org/licenses/by/4.0/).

how you have dealt with the points raised by the reviewers in the 'Response to Reviewers' box. If you do not agree with any of their criticisms or suggestions please explain clearly why this is so.

Reviewer 1

Advance summary and potential significance to field

Interesting paper investigating anti-neutrophil CDK9 inhibitors in promoting healing of damaged myocardium with an ultimate aim of treating heart attack patients with this strategy. The experiments are performed in zebrafish using a myocardial damage protocol developed by this group. The authors show that CDK9 inhibitors resolve myocardial inflammation and promote healing.

Comments for the author

The manuscript is generally well written and clear, although there are some areas where additional textual work would aid understanding and interpretation of the data.

The study is exclusively in zebrafish. Some context from other model systems would aid interpretation of the data - what evidence is there that neutrophils are involved in inhibition of carduiomyocyte proliferation and myocardial wound regression (line 89-90). How similar is the temporal response they have seen (lines 95-97).

The authors show that AT7519 is a selective CDK9 inhibitor in zebrafish. The text should be amended in several places to not make claims beyond where their studies end. E.g. Lines 106, 229, 270, 1060

The model used is not discussed at all anywhere in the manuscript, including in the methods. Although the method is published, it would greatly aid readers (not to mention reviewers) if they could understand what has been done here without having to find and read another manuscript. It would aid clarity if the figures could also include an outline showing the injury methodology. There is also no methodological detail provided for how wound area is quantified, with than it is done in ImageJ.

Line 127 - neutrophil numbers had mostly resolved… Can a number resolve? Neutrophil numbers had reduced to baseline is perhaps better? Elsewhere the manuscript refers to neutrophil resolution. Can a neutrophil resolve?
Line 131 -swarming behaviour is a very characteristic form of neutrophil behaviour that goes beyond just accumulating in one site. What evidence do the authors have that this is swarming and not just accumulation at sites of tissue injury? Again another paper is cited, but this could be briefly explained here.
Line 138 - the evidence that this is not apoptosis is a little unclear. The authors show that reverse migration occurs, but it does not mean that apoptosis does not occur, unless they have examined apoptosis (not shown?) or can account for every neutrophil. I was not clear if the latter was the case, from the data shown.
Line 151 - “macrophage recruitment was unaffected.” - macrophage number was assessed, not recruitment.

Paragraph starting line 162. This is concerning about the non-specific toxicity of this experimental setup.

Toxicity is evaluated by measuring EF and cardiomycocyte numbers. If this is a specific effect, these parameters should be unchanged in mutant animals. However, in the specificity experiments, these parameters are discarded in favour of heart rate, which is not assessed here. It would be better if the same assessment was done in each case. Some discussion should be added to clarify what the authors think is going on here. I am not convinced that this is not non-specific toxicity of these molecules. How do the doses compare to serum concentrations in treated humans?

© 2021. Published by The Company of Biologists under the terms of the Creative Commons Attribution License (https://creativecommons.org/licenses/by/4.0/).

Line 189 - no evidence is presented to show that neutrophil apoptosis is induced. Morphology of GFP positive cells is not an widely accepted readout of neutrophil apoptosis. The authors should tone down the language here or perform experiments to directly assess this.
Line 204 “retains cardiac macrophage presence” - can you retain a presence? Retain cardiac macrophage …might be better?
Line 212 and several other places - for compound transgenics ZFIN annotation requires the following notation: . Tg(mpeg1:mCherry)allelecode; Tg(TNFa:GFP)allelecode
https://zfin.atlassian.net/wiki/spaces/general/pages/1818394635/ZFIN+Zebrafish+Nomenclature+Conventions?focusedCommentId=99221554

Line 221 - I recommend removing the words: “is injury-specific, as the same phenotype”
Line 251 - is reduced heart rate really secondary to increased mortality? It would seem that it is either the other way round, or non-specific toxicity is driving both.
Line 279 - is turnover really increased? If there are more cells, there are many ways to deliver this, some of which include reduced turnover.

Most figures would be much improved by a diagram showing where the imaged areas are on the fish. The colour scale for time changes is a nice idea, but by starting with black, data is lost, as this is the same colour as the background. This also applies in other figures.

Reviewer 2

Advance summary and potential significance to field

In this manuscript Kaveh et al use their recently published laser injury model in zebrafish to study the innate immune response to cardiac injury. In specific they use 2 cdk9 inhibitors to target the neutrophil response. Using heartbeat synchronised light sheet microscopy they are able to clearly visualise the immune cells and to follow these over time. Prolonged exposure to the inhibitors results in increased reverse migration of neutrophils and macrophages, but also has adverse effects on the heart. Shorter exposure has no adverse effects and no influence on macrophage numbers, while treatment with AT7519 upregulates tnf in macrophages. They then go on to show that AT7519 is more selective than FVP followed by showing that short AT7519 treatment increases cardiomyocyte number and regeneration speed.

As knowledge on the neutrophil response during fish heart regeneration is still limited, this study provides interesting novel insights into this process.
However, the main findings are not investigated in enough depth to be significant and novel enough to the field to warrant publication in a journal like Development.

Comments for the author

My main comments that need addressing are:
- Figure 2D-E shows that prolonged treatment with both inhibitors results in reduced cardiomyocyte numbers, which is attributed to impaired cardiomyocyte turnover, however, this is not supported by experiments and needs further investigation. Is this caused by reduced proliferation, increased cell death? And is this the result of the reduction in neutrophil/macrophage numbers or a direct effect of the inhibitor on the cardiomyocytes? Cdk9 is expressed in many cell-types, including cardiomyocytes. Similar for 5C. This needs to be clarified.
- Figure 2-2: Is the reduction in neutrophils in the heart a result of the overall neutropenia?
- Line 368: “By limiting the CDK9i treatment period to a two-hour window we were able to enhance neutrophil resolution while avoiding all adverse effects.” Cardiac macrophage numbers are shown in response to the shorter treatment duration, but the cardiac neutrophil response needs to be shown as well. As the inhibitors are used to target neutrophil response, why does the remainder of the study not include neutrophils?
- Selectivity of AT7519 is only assessed by heart rate, what about gene expression and neutrophil/macrophage numbers?
- Lines 273-283: It is suggested that macrophage tnf upreglation following AT7519 treatment influences cardiomyocyte turnover. This conclusion can not be drawn based on the performed experiments, but these are experiments that should be performed to support the findings and to

© 2021. Published by The Company of Biologists under the terms of the Creative Commons Attribution License (https://creativecommons.org/licenses/by/4.0/).

increase novelty. For example, to show if increased tnf signalling has the same effect on cardiomyocyte number during embryonic heart regeneration. And does the reduction in neutrophils cause the upregulation of tnf in macrophages? The authors do acknowledge this issue and suggest possible experiments in lines 408-416.
- Lines 380-392: "Moreover, using LSFM timelapse imaging we directly observed a migratory TNF+ macrophage settle at the injury site, neighboring two wound-associated macrophages." This paragraph is based on the observation of one cell?

Minor comments:
- In Figure 1 D, the difference between using AT7519 and DMSO is clearly visible in the injury site, but the overall number of neutrophils in the AT7519 treated embryo seems higher. This needs further clarification. Is the number of neutrophils outside the heart higher at this time point? Or do they move around more?
- Figure 2-2D, neutrophil apoptosis needs to be confirmed by Tunel.
- The conclusions in general and particularly in the discussion need toning down.
- Were the experiments performed with randomisation and blinding?

Reviewer 3

Advance summary and potential significance to field

In this paper, Kaveh and colleagues used their established zebrafish larval cardiac injury model, combined with a bespoke live imaging system, to investigate whether the CDK9 inhibitors AT7519 and FVP can modulate larval cardiac regeneration. Since these two potent drugs have been widely used in clinical trials as anti-cancer therapies, it would be of great clinical relevance to find an FDA-approved immunomodulatory drug that could enhance cardiac regeneration after injury.
The authors analysed how neutrophil recruitment to the site of injury and consequent macrophage behaviour was affected in CDK9-inhibited injured larval hearts. They found that both AT7519 and FVP treatments resolved neutrophilic inflammation via reverse migration. Moreover, they found that transient treatment with AT7519, but not FVP, increased polarisation of wound-associated macrophages and accelerated the rate of myocardial wound closure in the zebrafish larva. To better understand the differential phenotypes observed with AT7519 or FVP treatment, the authors tested for Cdk9 selectivity of these inhibitors in vivo by using a cdk9 mutant line previously generated by Hoodless et al., 2016. They found that FVP displayed significant off-target effects, while AT7519 proved to be a selective CDK9 inhibitor.

Comments for the author

The finding of an FDA-approved immunomodulatory drug that can enhance cardiac regeneration after injury is of extreme clinical relevance. However, there are aspects of this study in its current form that reduce overall enthusiasm for it to be published in Development. This study is built on several findings made from the same group over the past years:
- The group has shown that both AT7519 and FVP drive neutrophil apoptosis in a CDK9-dependent manner to resolve inflammation following tail fin transection in larval zebrafish (Hoodless et al., 2016).
- They recently characterised neutrophil and macrophage migratory responses by in vivo imaging using the same larval zebrafish cardiac injury model (Taylor et al., 2019 and Kaveh et al., 2020). The authors showed in these studies that, following cardiac larval injury, there is an early acute phase of neutrophil recruitment, which is followed by sustained macrophage recruitment. They go on to show that after this initial recruitment, the innate immune response resolves by reverse migration, with very little apoptosis or efferocytosis of neutrophils.
- Importantly, the same group has shown that CDK9 and its repressor LARP7 modulate cardiomyocyte proliferation and response to injury in the zebrafish larval heart (Matrone et al., 2015 Journal of Cell Science).
Here the authors modulated CDK9 activity with FVP and injection of Cdk9- and Larp7-targeting morpholinos.
They showed that even a modest reduction of Cdk9 protein led to impaired cardiac structure and function reduced cardiomyocyte proliferation and, importantly, impaired functional recovery following cardiac laser injury. In contrast, enhancing Cdk9 activity through knockdown of its

© 2021. Published by The Company of Biologists under the terms of the Creative Commons Attribution License (https://creativecommons.org/licenses/by/4.0/).

repressor molecule, Larp7, increased cardiomyocyte proliferation and was associated with normal recovery of the ventricle from laser injury. Given the relevance of these results to the current study and the somehow contradictory findings, it comes as a surprise that Matrone et al 2015 is not cited or discussed by the authors in this manuscript.
Taken together, the nature of the findings presented in the current manuscript seems rather incremental.
Importantly, the authors have not established a direct mechanistic link between the CDK9-inhibition by AT7519 and the 1) associated neutrophil dispersion, 2) enhanced TNF expression in wound-associated macrophages and 3) acceleration of larval cardiac regeneration. Does CDK inhibition directly induce neutrophil reverse chemotaxis, which is then necessary for tnfa expression of wound-associated macrophages or is CDK9 directly promoting macrophage polarization? Is cardiomyocyte proliferation directly regulated by CDK9 inhibition or is it due to tnfa+ macrophage-derived mitogenic factors? Are all the reported events a cascade/consequence of each other or are they independently regulated by CDK9 inhibition?
Additionally, given the claimed specificity of the AT7519 drug, is the accelerated regenerative response observed in the larval injured heart reproduced in a more MI-relevant model, like the adult cryoinjured zebrafish heart (where the potential effect on scarring could also be assessed)? Or is the AT7519 effect on enhancing cardiomyocyte proliferation mainly favoured in a developmental/growth context?
For the above limitations, this reviewer does not think that the study, in its present form, offers a significant contribution to our understanding of the mechanism by which AT7519-driven CDK9 inhibition is accelerating cardiac regeneration.

A few other suggestions that the authors might consider addressing for a revised version of the manuscript are highlighted below:

- Is AT7519 promoting downregulation of a different set of primary inflammatory response genes, when compared to FVP-treated hearts? If so, could this be contributing to the differences in neutrophil and macrophage response observed when the injured heart is treated with these two different drugs?
- For clarity purposes, it would be useful to always include the myl7-GFP channel in the images and videos presented when injurying the Tg(myl7:GFP;mpx:mCherry) larvae, like the authors have done in Figure 1B. It makes it much clearer to the reader to understand where the heart muscle is and where the injury was performed. Would be important to indicate the injury area in all conditions. Arrows in Figure 1E and 1F should be consistent.
- Representative images of the data shown in Figure 2B graphs should be presented (similarly to what the authors did in Figure 1).
- For consistency purposes, the authors should consider always presenting the control condition/figure/video ahead of the manipulated/treated condition. For example, Supplementary Video 4 should represent the control condition and Supplementary Video 5 the FVP-treated heart.
- A more detailed description of the data presented in Supplemental Figures 1 and 2 would be advisable to 1) include the important study from the same group (Matrone et al., 2015) and 2) better describe the differences observed between injured and uninjured conditions, given that the data is presented but not mentioned in the results section.
- The authors claim that: “Unlike in mammalian models, only one macrophage polarisation marker has been reliably reported in larval zebrafish and this is TNF. These studies revealed TNF+ macrophages have pro-regenerative properties following spinal cord, somitic muscle and tail fin injury”. I would strongly recommend the authors to have a closer look at recent studies from the labs of Nadia Mercader and Rebecca Richardson, where wt1+ (Sanz-Morejón et al., 2019) and tnfα+ (Bevan et al., 2020) macrophage subpopulations have been characterised in the adult injured zebrafish heart and are relevant for this study.
- Line 89: the work by Kikuchi et al., 2010 should be added as a citation.
- Figure 2 – supplement 2: the embryos in DMSO and FVP seem to be at different magnifications - scale needs adjusting.

© 2021. Published by The Company of Biologists under the terms of the Creative Commons Attribution License (https://creativecommons.org/licenses/by/4.0/).

First revision

Author response to reviewers' comments

Development Reviewer's comments - point-by-point response

Reviewer 1 comments:

Reviewer 1 Advance Summary and Potential Significance to Field...

Interesting paper investigating anti-neutrophil CDK9 inhibitors in promoting healing of damaged myocardium, with an ultimate aim of treating heart attack patients with this strategy. The experiments are performed in zebrafish using a myocardial damage protocol developed by this group. The authors show that CDK9 inhibitors resolve myocardial inflammation and promote healing.

Reviewer 1 Comments for the Author...

The manuscript is generally well written and clear, although there are some areas where additional textual work would aid understanding and interpretation of the data.

The study is exclusively in zebrafish. Some context from other model systems would aid interpretation of the data - what evidence is there that neutrophils are involved in inhibition of cardiomyocyte proliferation and myocardial wound regression (line 89-90). How similar is the temporal response they have seen (lines 95-97).

We thank the reviewer for their suggestion and agree that more specific information regarding the role of neutrophils in heart regeneration would help interpretation of the results presented. As very little is known about the role of neutrophils in cardiac regeneration outside of the studies cited (Lai et al., 2017 and Xu et al., 2019), we have expanded on these (lines 90-92). The temporal dynamics of the immune response in the larval zebrafish cardiac laser injury model closely recapitulates that of adult zebrafish and murine models of heart injury. We agree that it would be beneficial to state this comparison and have done so accordingly (lines 98-100).

The authors show that AT7519 is a selective CDK9 inhibitor in zebrafish. The text should be amended in several places to not make claims beyond where their studies end. E.g. Lines 106, 229, 270, 1060

We thank the reviewer for bringing this to our attention, we have amended this accordingly to reflect that the findings were zebrafish-based.

The model used is not discussed at all anywhere in the manuscript, including in the methods. Although the method is published, it would greatly aid readers (not to mention reviewers) if they could understand what has been done here without having to find and read another manuscript. It would aid clarity if the figures could also include an outline showing the injury methodology. There is also no methodological detail provided for how wound area is quantified, with than it is done in ImageJ.

We thank the reviewer for raising this and although the characterisation of the laser injury model has been published (Kaveh et al., 2020), we agree that it would help readers to describe our injury model in more detail within the manuscript. As suggested, we have provided a new schematic outline (Figure 2-supplement 1A) to assist with understanding how the laser injury is performed. We have now also provided greater detail in the methodology describing the laser injury method (lines 518-531), in addition to how various analyses were performed, such as wound area quantification (lines 655-660).

Line 127 - neutrophil numbers had mostly resolved… Can a number resolve? Neutrophil numbers had reduced to baseline is perhaps better? Elsewhere the manuscript refers to neutrophil resolution. Can a neutrophil resolve?

© 2021. Published by The Company of Biologists under the terms of the Creative Commons Attribution License (https://creativecommons.org/licenses/by/4.0/).

We agree with the reviewer's suggestion and have amended the text to either reflect neutrophil numbers returning to baseline or neutrophilic inflammation resolving.

Line 131 -swarming behaviour is a very characteristic form of neutrophil behaviour that goes beyond just accumulating in one site. What evidence do the authors have that this is swarming and not just accumulation at sites of tissue injury? Again another paper is cited, but this could be briefly explained here.

We thank the reviewer for this suggestion. We have removed our description of "swarming behaviour" in this context as our timelapse imaging starts from 4 hpi, therefore the pioneer neutrophil has already been recruited to the injury site and secondary neutrophil recruitment/clustering has already begun (Supplementary Video 1). We have previously shown that neutrophils display swarm-like behaviour in our cardiac injury model (Kaveh et al., 2020) where timelapse imaging was started earlier than 2 hpi and pioneer neutrophil recruitment was observed to precede secondary neutrophil recruitment.

Line 138 - the evidence that this is not apoptosis is a little unclear. The authors show that reverse migration occurs, but it does not mean that apoptosis does not occur, unless they have examined apoptosis (not shown?) or can account for every neutrophil. I was not clear if the latter was the case, from the data shown.

Our real-time heartbeat-synchronised LSFM imaging approach allows us to account for and track all neutrophils recruited to the cardiac lesion (Taylor et al., 2019 and Kaveh et al., 2020). We thank the reviewer for raising this and have amended the results text accordingly to aid clarity (lines 143-146).

Line 151 - "macrophage recruitment was unaffected." - macrophage number was assessed, not recruitment.

We thank the reviewer for bringing this to our attention, we have now amended this text.

Paragraph starting line 162. This is about the non-specific toxicity of this experimental setup. Toxicity is evaluated by measuring EF and cardiomyocyte numbers. If this is a specific effect, these parameters should be unchanged in mutant animals. However, in the specificity experiments, these parameters are discarded in favour of heart rate, which is not assessed here. It would be better if the same assessment was done in each case. Some discussion should be added to clarify what the authors think is going on here. I am not convinced that this is not non-specific toxicity of these molecules. How do the doses compare to serum concentrations in treated humans?

We thank the reviewer for raising this. Heart rate was chosen as a surrogate for toxicity and to aid with high throughput evaluation of individual animals across short time scales.
Furthermore, a loss of cardiac function, specifically heart rate, is one of the first phenotypic readouts of drug-induced toxicity recognised in larval zebrafish (Rubinstein, 2006 and Kithcart and MacRae, 2017). We agree that it would be beneficial to report the effect of CDK9i treatment on heart rate and thus have now included this data (Figure 2-supplement 1C). Our data show that heart rate is reduced at 48 hpi with FVP in both injured and uninjured larvae, supporting the use of heart rate as a readout for toxicity in the selectivity model. We acknowledge that our selectivity assay may not differentiate cardiotoxicity from general toxicity.

Line 189 - no evidence is presented to show that neutrophil apoptosis is induced. Morphology of GFP positive cells is not an widely accepted readout of neutrophil apoptosis. The authors should tone down the language here or perform experiments to directly assess this.

We agree with the reviewer's suggestion and have toned down the language here.

Line 204 "retains cardiac macrophage presence" - can you retain a presence? Retain cardiac macrophage ...might be better?

We agree with the reviewer's suggestion and have edited this sentence accordingly to reflect macrophage numbers as opposed to presence.

© 2021. Published by The Company of Biologists under the terms of the Creative Commons Attribution License (https://creativecommons.org/licenses/by/4.0/).

Line 212 and several other places - for compound transgenics ZFIN annotation requires the following anotation: Tg(mpeg1:mCherry)allelecode; Tg(TNFa:GFP)allelecode

https://zfin.atlassian.net/wiki/spaces/general/pages/1818394635/ZFIN+Zebrafish+Nomenclature+Conventions?focusedCommentId=99221554

We thank the reviewer for spotting this. We have now ensured that all allele codes are included for all zebrafish lines cited in the methods section.

Line 221 - I recommend removing the words: “is injury-specific, as the same phenotype”

We agree with the reviewer’s suggestion and have edited this sentence accordingly.

Line 251 - is reduced heart rate really secondary to increased mortality? It would seem that it is either the other way round, or non-specific toxicity is driving both.

We agree with the reviewer that this sentence is rather confusing as one is probably not completely secondary of the other. As such we have amended these sentences.

Line 279 - is turnover really increased? If there are more cells, there are many ways to deliver this, some of which include reduced turnover.

We agree that “turnover” is not the most accurate description of this finding and have edited this accordingly throughout the manuscript to reflect cardiomyocyte “numbers”.

Most figures would be much improved by a diagram showing where the imaged areas are on the fish. The colour scale for time changes is a nice idea, but by starting with black, data is lost, as this is the same colour as the background. This also applies in other figures.

We thank the reviewer for these suggestions. As most of our figures almost occupy a full A4 page, we have struggled to incorporate these diagrams without overcrowding the figures.

We have made sure to clarify in the figure legends exactly which region larvae were imaged (pericardium, heart, caudal hematopoietic tissue, head or whole body).
Regarding the temporal colour code, although a short duration of immune cell migration will be depicted as a darker colour tone, we believe that these images represent an accurate summary of immune cell activity on the injured heart (accumulation vs reverse migration).

Reviewer 2 comments:

Reviewer 2 Advance Summary and Potential Significance to Field...

In this manuscript Kaveh et al use their recently published laser injury model in zebrafish to study the innate immune response to cardiac injury. In specific, they use 2 cdk9 inhibitors to target the neutrophil response. Using heartbeat synchronised light sheet microscopy they are able to clearly visualise the immune cells and to follow these over time. Prolonged exposure to the inhibitors results in increased reverse migration of neutrophils and macrophages, but also has adverse effects on the heart. Shorter exposure has no adverse effects and no influence on macrophage numbers, while treatment with AT7519 upregulates tnf in macrophages. They then go on to show that AT7519 is more selective than FVP, followed by showing that short AT7519 treatment increases cardiomyocyte number and regeneration speed.

As knowledge on the neutrophil response during fish heart regeneration is still limited, this study provides interesting novel insights into this process.
However, the main findings are not investigated in enough depth to be significant and novel enough to the field to warrant publication in a journal like Development.

© 2021. Published by The Company of Biologists under the terms of the Creative Commons Attribution License (https://creativecommons.org/licenses/by/4.0/).

Reviewer 2 Comments for the Author...

My main comments that need addressing are:

- Figure 2D-E shows that prolonged treatment with both inhibitors results in reduced cardiomyocyte numbers, which is attributed to impaired cardiomyocyte turnover, however, this is not supported by experiments and needs further investigation. Is this caused by reduced proliferation, increased cell death? And
is this the result of the reduction in neutrophil/macrophage numbers or a direct effect of the inhibitor on the cardiomyocytes? Cdk9 is expressed in many cell- types, including cardiomyocytes. Similar for 5C. This needs to be clarified.

We agree with the reviewer that the use of "cardiomyocyte turnover" requires clarification. As also advised by reviewer 1, we have now edited cardiomyocyte "turnover" to "numbers" throughout the manuscript. Our laboratory has previously shown that continuous FVP treatment reduces cardiomyocyte numbers by specifically inhibiting cardiomyocyte proliferation in larval zebrafish (Matrone et al., 2015). In this manuscript we have observed a similar reduction in cardiomyocyte numbers with continuous FVP and AT7519 treatments, suggesting that the same anti-proliferative effect could be occurring, although we acknowledge that cardiomyocyte apoptosis could be contributing to the reduction in cardiomyocyte numbers. This is now mentioned in the discussion.

The reviewer raises an interesting point regarding the loss of cardiac-recruited neutrophils/macrophages contributing to the reduction in cardiomyocyte numbers. In uninjured control hearts the number of neutrophils or macrophages is unaffected by CDK9i treatment as there are very few immune cells on the heart (Figures 1 and 2). This suggests that neutrophils and macrophages are not reducing cardiomyocyte numbers during steady state. In cardiac injury, macrophages are widely implicated as important regulators of cardiac regeneration, and our data support this also. When applying the transient (pulsed) CDK9i treatment, we found that reduced neutrophil numbers did not affect macrophage recruitment/retention, ejection fraction, and AT7519 (but not FVP) enhanced cardiomyocyte number expansion (Figure 5), which was associated with augmented macrophage *tnf* polarisation (Figure 3). We have now taken this further by investigating the role of macrophages in the AT7519-associated increase in cardiomyocyte numbers following injury. Our newly acquired data (Figure 6) show that macrophage-null larval zebrafish do not exhibit an injury-associated increase in cardiomyocyte numbers following transient AT7519 treatment, whereas their wild-type treated counterparts do, as also shown originally (Figure 5). These data suggest that macrophages are required for the enhanced cardiomyocyte regenerative response following AT7519 treatment. We have revised the text in the results and discussion sections accordingly to include these points.

- Figure 2-2: Is the reduction in neutrophils in the heart a result of the overall neutropenia?

We found neutropenia to be apparent only at 48 hpi (Figure 2 – Supplement 2B) whereas the reduction in cardiac-recruited neutrophils was present at 6 hpi (Figure 1C), hence we can rule this out.

- Line 368: "By limiting the CDK9i treatment period to a two-hour window, we were able to enhance neutrophil resolution while avoiding all adverse
effects." Cardiac macrophage numbers are shown in response to the shorter treatment duration, but the cardiac neutrophil response needs to be shown as well. As the inhibitors are used to target neutrophil response, why does the remainder of the study not include neutrophils?

We thank the reviewer for raising this. We had already performed the transient treatment experiment using *Tg(myl7:GFP;mpx:mCherry)* larvae with both AT7519 and FVP (as performed initially in Figure 1) and have now included this data (Figure 3-supplement 1B). Our data confirms that transient treatment with AT7519 and FVP resolves neutrophilic inflammation at 6 hpi, which returns to control levels by 24 hpi.

- Selectivity of AT7519 is only assessed by heart rate, what about gene expression and

© 2021. Published by The Company of Biologists under the terms of the Creative Commons Attribution License (https://creativecommons.org/licenses/by/4.0/).

neutrophil/macrophage numbers?

We thank the reviewer for this comment. We have now included additional data supporting the use of heart rate as a high throughput read out of toxicity in this model (Figure 2- supplement 1C). Furthermore, a loss of cardiac function, specifically heart rate, is one of the first phenotypic readouts of drug-induced toxicity recognised in larval zebrafish (Rubinstein, 2006 and Kithcart and MacRae, 2017). Although potentially more insightful, gene expression as a drug toxicity readout would be difficult to perform for individual animals across short time scales, as several larvae would need to be pooled for qPCR or RNA sequencing. With regards to assessing neutrophil/macrophage numbers, *cdk9* homozygous mutants are severely neutropenic and fewer neutrophils and macrophages are recruited following wounding (Hoodless et al., 2016), thus hindering their assessment. Taken together we believe heart rate to be one of the most suitable read outs of drug-induced toxicity in larval zebrafish. We have included additional text in the discussion highlighting the pros and cons of our selectivity assay, as described above.

- Lines 273-283: It is suggested that macrophage tnf upreglation following AT7519 treatment influences cardiomyocyte turnover. This conclusion can not be drawn based on the performed experiments, but these are experiments that should be performed to support the findings and to increase novelty. For example, to show if increased tnf signalling has the same effect on cardiomyocyte number during embryonic heart regeneration. And does the reduction in neutrophils cause the upregulation of tnf in macrophages? The authors do acknowledge this issue and suggest possible experiments in lines 408-416.

We thank the reviewer for their experimental suggestions to further increase the novelty of our manuscript. As mentioned above, we have now performed additional experiments using macrophage-null zebrafish and found that macrophages are required for the improved regenerative response following AT7519 treatment (Figure 6). However, we acknowledge that our findings do not completely explore the role of macrophage-derived *tnf* in this enhanced regenerative response. Regarding this, we wish to refer to two recent and highly relatable publications (Cavone et al., 2021 and Ratnayake et al., 2021) to expand on the pro-regenerative *tnf*$^{+}$ macrophage subpopulation points further, as also mentioned to reviewer 3. In both studies, single-cell RNA sequencing of wound-recruited macrophages was performed following larval zebrafish injury.

1. Developmental Cell paper by Cavone et al. (2021) found that the spinal cord lesion *tnf*$^{+}$ macrophage subpopulation significantly upregulates genes encoding for mitogenic factors, namely *hbegf* and *tnf* itself. This *tnf*$^{+}$ macrophage subpopulation is specifically required and sufficient for spinal cord progenitor cell proliferation and regeneration.
2. Nature paper by Ratnayake et al. (2021) similarly found that a subpopulation of wound musculature-dwelling macrophages secrete *nampt* to promote satellite cell proliferation and muscle regeneration.

We have now referred to the scRNA-seq metadata provided in both studies and identify shared mitogenic genes (specifically *tnf* and *hbegf*) upregulated in both pro-regenerative macrophage subpopulations and not in any other macrophage subpopulation/cluster. This further suggests that the *tnf*$^{+}$ macrophage polarisation shown to be augmented by AT7519 in our study could be driving the enhanced cardiomyocyte regenerative response. However, whilst *tnf* macrophages appear to be a pro-regenerative subpopulation in larval zebrafish (Nguyen-Chi et al., 2017; Tsarouchas et al., 2018; Gurevich et al., 2018 and Cavone et al., 2021), *tnf* is one of many differentially regulated growth factors/cytokines in these cells that could be promoting cardiomyocyte regeneration. Hence testing and characterising all of these differentially regulated genes in our model by performing high resolution transcriptomic experiments and analysis is ultimately beyond the scope of this study. We have, however, now referred to these points in the discussion.

- Lines 380-392: “Moreover, using LSFM timelapse imaging we directly observed a migratory TNF+ macrophage settle at the injury site, neighboring two wound-associated macrophages.” This paragraph is based on the observation of one cell?

We acknowledge that this is a single observation and have now removed the associated text.

Minor comments:

- In Figure 1 D, the difference between using AT7519 and DMSO is clearly visible in the

© 2021. Published by The Company of Biologists under the terms of the Creative Commons Attribution License (https://creativecommons.org/licenses/by/4.0/).

injury site, but the overall number of neutrophils in the AT7519 treated embryo seems higher. This needs further clarification. Is the number of neutrophils outside the heart higher at this time point? Or do they move around more?

The reviewer is quite right to suggest that superficially neutrophil numbers seem higher in the AT7519-treated heart presented in Figure 1D. We would like to make clear that this image is derived from timelapse data (between 4 hpi and 6 hpi) and has been temporally colour coded. Thus, neutrophil positions appear as a different colour depending on the point in time (as indicated in the figure key). This allows neutrophil migration between timepoints to be summarised and represented in one image. As such, the AT7519-treated fish in Figure 1D represents neutrophils migrating more erratically on the injured heart. As indicated with arrowheads (Figure 1F), neutrophils eventually reverse migrate in the presence of AT7519 (as opposed to being specifically retained at the ventricular apex injury site in the presence of DMSO, Figure 1D/E). As this was not completely clear, we have amended the results text and figure legend accordingly to aid interpretation of this data.

- Figure 2-2D, neutrophil apoptosis needs to be confirmed by Tunel.

We agree with the reviewer's suggestion that in order to confirm neutrophil apoptosis TUNEL staining would be required. As also advised by reviewer 1, we have amended the text accordingly to tone down the conclusions drawn.

- The conclusions in general and particularly in the discussion need toning down.

We acknowledge that in certain sections, particularly the discussion, the text could be toned down and have done so accordingly.

- Were the experiments performed with randomisation and blinding?

At the start of each experiment, larvae were screened for the relevant fluorescent signals and then randomly allocated to different experimental groups. All analysis was performed blinded to treatment groups. We thank the reviewer for raising this and have now included this statement in the methods section.

Reviewer 3 comments:

Reviewer 3 Advance Summary and Potential Significance to Field...

In this paper, Kaveh and colleagues used their established zebrafish larval cardiac injury model, combined with a bespoke live imaging system, to investigate whether the CDK9 inhibitors AT7519 and FVP can modulate larval cardiac regeneration. Since these two potent drugs have been widely used in clinical trials as anti-cancer therapies, it would be of great clinical relevance to find an FDA-approved immunomodulatory drug that could enhance cardiac regeneration after injury. The authors analysed how neutrophil recruitment to the site of injury and consequent macrophage behaviour was affected in CDK9-inhibited injured larval hearts. They found that both AT7519 and FVP treatments resolved neutrophilic inflammation via reverse migration. Moreover, they found that transient treatment with AT7519, but not FVP, increased polarisation of wound- associated macrophages and accelerated the rate of myocardial wound closure in the zebrafish larva. To better understand the differential phenotypes observed with AT7519 or FVP treatment, the authors tested for Cdk9 selectivity of these inhibitors in vivo by using a cdk9 mutant line previously generated by Hoodless et al., 2016. They found that FVP displayed significant off-target effects, while AT7519 proved to be a selective CDK9 inhibitor.

Reviewer 3 Comments for the Author...

The finding of an FDA-approved immunomodulatory drug that can enhance cardiac regeneration after injury is of extreme clinical relevance. However, there are aspects of this study in its current form that reduce overall enthusiasm for it to be published in Development. This study is built on

© 2021. Published by The Company of Biologists under the terms of the Creative Commons Attribution License (https://creativecommons.org/licenses/by/4.0/).

several findings made from the same group over the past years:
- The group has shown that both AT7519 and FVP drive neutrophil apoptosis in a CDK9-dependent manner to resolve inflammation following tail fin transection in larval zebrafish (Hoodless et al., 2016).
- They recently characterised neutrophil and macrophage migratory responses by in vivo imaging using the same larval zebrafish cardiac injury model (Taylor et al., 2019 and Kaveh et al., 2020). The authors showed in these studies that, following cardiac larval injury, there is an early acute phase of neutrophil recruitment, which is followed by sustained macrophage recruitment. They go on to show that after this initial recruitment, the innate immune response resolves by reverse migration, with very little apoptosis or efferocytosis of neutrophils.

Importantly, the same group has shown that CDK9 and its repressor LARP7 modulate cardiomyocyte proliferation and response to injury in the zebrafish larval heart (Matrone et al., 2015 Journal of Cell Science). Here the authors modulated CDK9 activity with FVP and injection of Cdk9- and Larp7-targeting morpholinos. They showed that even a modest reduction of Cdk9 protein led to impaired cardiac structure and function, reduced cardiomyocyte proliferation and, importantly, impaired functional recovery following cardiac laser injury. In contrast, enhancing Cdk9 activity through knockdown of its repressor molecule, Larp7, increased cardiomyocyte proliferation and was associated with normal recovery of the ventricle from laser injury. Given the relevance of these results to the current study and the somehow contradictory findings, it comes as a surprise that Matrone et al 2015 is not cited or discussed by the authors in this manuscript.

Taken together, the nature of the findings presented in the current manuscript seems rather incremental.

Importantly, the authors have not established a direct mechanistic link between the CDK9-inhibition by AT7519 and the 1) associated neutrophil dispersion, 2) enhanced TNF expression in wound-associated macrophages and 3) acceleration of larval cardiac regeneration. Does CDK inhibition directly induce neutrophil reverse chemotaxis, which is then necessary for tnfa expression of wound- associated macrophages or is CDK9 directly promoting macrophage polarization? Is cardiomyocyte proliferation directly regulated by CDK9 inhibition or is it due to tnfa+ macrophage-derived mitogenic factors? Are all the reported events a cascade/consequence of each other or are they independently regulated by CDK9 inhibition? Additionally, given the claimed specificity of the AT7519 drug, is the accelerated regenerative response observed in the larval injured heart reproduced in a more MI-relevant model, like the adult cryoinjured zebrafish heart (where the potential effect on scarring could also be assessed)? Or is the AT7519 effect on enhancing cardiomyocyte proliferation mainly favoured in a developmental/growth context? For the above limitations, this reviewer does not think that the study, in its present form, offers a significant contribution to our understanding of the mechanism by which AT7519- driven CDK9 inhibition is accelerating cardiac regeneration.

We would like to thank the reviewer for their interpretation and suggestions for further work. We agree that discussing the findings from Matrone et al. (2015) would be beneficial as the results from our study are complementary. As shown by Matrone et al. (2015), we also found that continuous pharmacological CDK9i treatment (in this manuscript with both FVP and AT7519) is detrimental for cardiac development and injury-associated regeneration in larval zebrafish (reduction in cardiac function and cardiomyocyte numbers). Matrone et al. (2015) found that continuous FVP treatment reduces cardiomyocyte numbers specifically by inhibiting cardiomyocyte proliferation in larval zebrafish. In this manuscript we observed a similar reduction in cardiomyocyte numbers and ejection fraction with continuous FVP and AT7519 treatments, suggesting the same anti-proliferative effect could be occurring, although we acknowledge that cardiomyocyte apoptosis could be contributing to the reduction in cardiomyocyte numbers. We have provided a summary of the above points in the discussion.

We respectfully disagree with the reviewer and believe the nature of these findings are more than incremental. The aim of this study was to examine if we could therapeutically enhance the resolution of neutrophilic inflammation and if so, what impact this would have on cardiomyocyte regeneration. Our study is the first to show that an FDA approved drug can improve cardiomyocyte regeneration via an immunomodulatory mechanism (please see following response for new mechanistic data). Furthermore, we have provided novel timelapse imaging of inflammatory cell behaviour and myocardial regeneration, in addition to proposing a novel assay to infer drug

© 2021. Published by The Company of Biologists under the terms of the Creative Commons Attribution License (https://creativecommons.org/licenses/by/4.0/).

selectivity in vivo.

We thank the reviewer for raising the mechanistic links between the immune cell and cardiomyocyte regeneration cascade and agree that it would benefit from further clarification. Our data indicate that selective or non-selective pharmacological CDK9 inhibition (i.e., with AT7519 or FVP) induces neutrophil reverse migration. As AT7519 later promotes macrophage *tnf* polarisation (but FVP does not), we postulate this is due to the higher degree of compound selectivity that AT7519 exhibits (Figure 4). We would like to further explain why we believe the enhanced regenerative response to be macrophage dependent. We have now performed additional experiments to determine the involvement of macrophages during the enhanced cardiomyocyte number expansion following transient AT7519 treatment. Using macrophage-null zebrafish we found that macrophages are required for the improved regenerative response following AT7519 treatment (Figure 6). However, we acknowledge that our findings do not fully explore the role of macrophage- derived *tnf* in this enhanced regenerative response. Our laboratory group has attempted performing bulk RNA sequencing on cardiac-recruited macrophages. However, due to the relatively low number of immune cells recruited to the larval heart following injury, we are unable to obtain a sufficient yield of isolated hearts to perform the experiment on isolated (FACS sorted) macrophages. As such, we wish to refer to two recent and highly relatable publications (Cavone et al., 2021 and Ratnayake et al., 2021) to expand on the pro- regenerative *tnf*$^+$ macrophage subpopulation points further. In both studies, single-cell RNA sequencing of wound-recruited macrophages was performed following larval zebrafish injury.

1. Developmental Cell paper by Cavone et al. (2021) found that the spinal cord lesion *tnf*$^+$ macrophage subpopulation significantly upregulates genes encoding for mitogenic factors, namely *hbegf* and *tnf* itself. This *tnf*$^+$ macrophage subpopulation is specifically required and sufficient for spinal cord progenitor cell proliferation and regeneration.
2. Nature paper by Ratnayake et al. (2021) similarly found that a subpopulation of wound musculature-dwelling macrophages secrete *nampt* to promote satellite cell proliferation and muscle regeneration.

We have now referred to the scRNA-seq metadata provided in both studies and identify shared mitogenic genes (specifically *tnf* and *hbegf*) upregulated in both pro-regenerative macrophage subpopulations and not in any other macrophage subpopulation/cluster. This further suggests that the *tnf*$^+$ macrophage polarisation shown to be augmented by AT7519 in our study could be driving the enhanced cardiomyocyte regenerative response. However, whilst *tnf* macrophages appear to be a pro-regenerative subpopulation in larval zebrafish (Nguyen-Chi et al., 2017; Tsarouchas et al., 2018; Gurevich et al., 2018 and Cavone et al., 2021), *tnf* is one of many differentially regulated growth factors/cytokines in these cells that could be promoting cardiomyocyte regeneration. Hence testing and characterising all of these differentially regulated genes in our model by performing high resolution transcriptomic experiments and analysis is ultimately beyond the scope of this study. We have, however, now referred to these points in the discussion.

As the induction of neutrophil reverse migration occurs before *tnf* polarisation of macrophages and cardiomyocyte regeneration, we postulate that the early CDK9-dependent resolution of neutrophilic inflammation promoted by AT7519 triggers the downstream regenerative cascade. As mentioned, our new data also support that this improved regenerative response with AT7519 is a macrophage dependent process.

We acknowledge that our larval zebrafish cardiac injury model may not exhibit scarring to the same extent as adult zebrafish cardiac cryoinjury and that it would be interesting to assess cardiac fibrosis in the adult model. However, in the current climate where lab restrictions are still partially imposed at our institute, performing equivalent adult zebrafish cardiac cryoinjury experiments would take at least 12 months. Unlike adult zebrafish, larval zebrafish are small, transparent and easy to handle, making them ideal for live imaging screening studies. Furthermore, it can take up to three months for zebrafish to reach adulthood, and due to difficulties with live imaging of immune cells at adult stages, experimentation often involves surgery, tissue fixation and antibody staining. These procedures are much more time consuming and heart regeneration takes at least two months in adult zebrafish; thus, statistical power is much harder to achieve in a timely manner. Therefore, we believe adult zebrafish and mouse experimentation should form a separate body of work. As sustained neutrophil retention has been shown to inhibit cardiomyocyte proliferation, promote cardiomyocyte apoptosis and delay scar regression following adult zebrafish

© 2021. Published by The Company of Biologists under the terms of the Creative Commons Attribution License (https://creativecommons.org/licenses/by/4.0/).

cardiac cryoinjury (Xu et al., 2019), we would expect timely AT7519 treatment to have a similar immunomodulatory and pro-regenerative effect in this model.

We have provided data to support that the effect of AT7519 is cardiac injury specific rather than developmental/growth related. When we apply the transient treatment to uninjured larvae, we do not observe an increase in cardiac *tnf*$^{+}$ macrophage presence or cardiomyocyte number expansion (Figure 3-supplement 2 and Figure 5-supplement 1). This injury-specific effect of increased *tnf*$^{+}$ macrophage numbers was also described by Cavone et al. (2021). We have more clearly highlighted the above points in the results and discussion sections of the manuscript.

A few other suggestions that the authors might consider addressing for a revised version of the manuscript are highlighted below:

- Is AT7519 promoting downregulation of a different set of primary inflammatory response genes, when compared to FVP-treated hearts? If so, could this be contributing to the differences in neutrophil and macrophage response observed when the injured heart is treated with these two different drugs?

The reviewer raises an interesting and relevant point here. Our selectivity data suggests that FVP could be inhibiting additional injury response genes that AT7519 does not through its more CDK9-dependent mechanism of action (Figure 4). To determine which additional injury response genes FVP may be inhibiting, RNA sequencing of isolated and pooled injured hearts could be performed. We have added this as a discussion point.

- For clarity purposes, it would be useful to always include the myl7-GFP channel in the images and videos presented when injurying the Tg(myl7:GFP;mpx:mCherry) larvae, like the authors have done in Figure 1B. It makes it much clearer to the reader to understand where the heart muscle is and where the injury was performed. Would be important to indicate the injury area in all conditions. Arrows in Figure 1E and 1F should be consistent.

We thank the reviewer for these suggestions. In some of the timelapse datasets presented the *myl7:GFP* transgene was not present, and so the outline of the heart was identified and indicated using GFP autofluorescence. We have now edited figure panels to indicate the site of laser injury with white arrowheads. We have also normalised the size of the arrowheads in Figure 1E and 1F (white arrowhead outline indicates neutrophil reference point in 1D, as stated in the figure legend).

- Representative images of the data shown in Figure 2B graphs should be presented (similarly to what the authors did in Figure 1).

We thank the reviewer for this suggestion. As we wanted to depict macrophage wound accumulation and migration with CDK9i treatment, we opted to use the LSFM timelapse- derived image panels here (as also shown in Figure 1E and 1F), which is not possible with the epifluorescence-based image panels.

- For consistency purposes, the authors should consider always presenting the control condition/figure/video ahead of the manipulated/treated condition. For example, Supplementary Video 4 should represent the control condition and Supplementary Video 5 the FVP-treated heart.

We thank the reviewer for spotting this. We have now ensured that the DMSO control video is placed before the FVP-treated video.

- A more detailed description of the data presented in Supplemental Figures 1 and 2 would be advisable to 1) include the important study from the same group (Matrone et al., 2015) and 2) better describe the differences observed between injured and uninjured conditions, given that the data is presented but not mentioned in the results section.

We thank the reviewer for these suggestions. As mentioned in our first response, we have now included a comparison of our findings to Matrone et al. (2015), highlighting that the continuous

© 2021. Published by The Company of Biologists under the terms of the Creative Commons Attribution License (https://creativecommons.org/licenses/by/4.0/).

CDK9i treatment data are similar. We have now expanded on the associated textual points for Figure 2-supplement 1 by including new data showing the effect of FVP on heart rate.

- The authors claim that: “Unlike in mammalian models, only one macrophage polarisation marker has been reliably reported in larval zebrafish and this is TNF. These studies revealed TNF+ macrophages have pro-regenerative properties following spinal cord, somitic muscle and tail fin injury”. I would strongly recommend the authors to have a closer look at recent studies from the labs of Nadia Mercader and Rebecca Richardson, where wt1+ (Sanz-Morejón et al., 2019) and tnfα+ (Bevan et al., 2020) macrophage subpopulations have been characterised in the adult injured zebrafish heart and are relevant for this study.

We thank the reviewer for these suggestions. We would like to highlight that here we are referring to larval zebrafish studies as opposed to adult, but the reviewer is quite right to point out that the adult studies are highly relevant. Bevan et al. (2020) found that *tnf*$^{+}$ macrophages prolong scar retention, whereas *tnf*$^{-}$ macrophages promote scar removal during regeneration. Cavone et al. (2021) demonstrated that *tnf*$^{+}$ macrophages have a pro- proliferative role following injury in larval zebrafish, suggesting a transition in *tnf* macrophage function during zebrafish development. We have now included these points and cited the Sanz-Morejon et al. (2019) paper in the discussion.

- Line 89: the work by Kikuchi et al., 2010 should be added as a citation.

We thank the reviewer for their suggestion and have now included this citation.

- Figure 2 – supplement 2: the embryos in DMSO and FVP seem to be at different magnifications - scale needs adjusting.

We thank the reviewer for spotting this, we have now corrected the scale bar length.

<u>Second decision letter</u>

MS ID#: DEVELOP/2021/199636

MS TITLE: Selective CDK9 inhibition resolves neutrophilic inflammation and enhances cardiac regeneration in larval zebrafish

AUTHORS: Aryan Kaveh, Finnius A Bruton, Magdalena E M Oremek, Carl S Tucker, Jonathan M Taylor, John J Mullins, Adriano G Rossi, and Martin A Denvir
ARTICLE TYPE: Research Article

I am happy to tell you that your manuscript has been accepted for publication in Development, pending our standard ethics checks. You may like to take note of one of the reviewer's comments about fish nomenclature but this can be dealt with at proof stage.

<u>Reviewer 1</u>

Advance summary and potential significance to field

as previous review

Comments for the author

The authors have satisfactory addressed my comments.
There is still an error with the way that double transgenics are reported. The authors use Tg(mpeg1:mCherry;
TNFa:GFP)allelecode, but the correct way to report this is Tg(mpeg1:mCherry)allelecode;

© 2021. Published by The Company of Biologists under the terms of the Creative Commons Attribution License (https://creativecommons.org/licenses/by/4.0/).

Tg(TNFa:GFP)allelecode .
Many authors (myself included) do not always get this right, and many journals do not police it. So, I leave this with the authors to decide if they wish to set a good example for the field!

Reviewer 2

Advance summary and potential significance to field

In my previous review, I have raised my concern that the main findings are not investigated in enough depth to be significant and novel enough to the field to warrant publication in a journal like Development, and indicated that additional experiments were required to increase the novelty of the paper. However, except for the nice addition of the macrophage null data, I feel the authors have mainly made textual changes to refer to other papers, clarify or tone down the findings.
In my opinion this has not raised the significance and novelty of the paper to the required level. However, while I question the level of novelty, the findings in the manuscript are robust, novel and of interest and I do realise that the past year has not made it easy to perform many additional experiments.
Eventually, it is not my role to decide if this is enough for publication.

Comments for the author

I do not have additional comments

Reviewer 3

Advance summary and potential significance to field

The revised manuscript clarifies pending concerns and questions from the reviewers. In particular, the authors have performed additional experiments using macrophage-null zebrafish and found that macrophages are required for the enhanced regenerative response upon AT7519 treatment. Although the authors do not experimentally address the role of macrophage-derived tnf in such regenerative response they have drawn upon two recent publications which explore the role of pro-regenerative tnf+ macrophages in other regenerative contexts (Cavone et al., 2021 and Ratnayake et al., 2021) to further support their claims.
Overall, the authors have addressed comments satisfactorily and I recommend this manuscript for publication in its current form.

Comments for the author

There are no further revisions necessary.

© 2021. Published by The Company of Biologists under the terms of the Creative Commons Attribution License (https://creativecommons.org/licenses/by/4.0/).
